# The Study on the Mechanism of Hugan Tablets in Treating Drug-Induced Liver Injury Induced by Atorvastatin

**DOI:** 10.3389/fphar.2021.683707

**Published:** 2021-06-28

**Authors:** Shujing Lv, Honghong Yu, Xinyue Liu, Xiaoyan Gao

**Affiliations:** School of Chinese Materia Medica, Beijing University of Chinese Medicine, Beijing, China

**Keywords:** Hugan tablets, drug-induced liver injury, UPLC-Q-TOF-MS/MS, network pharmacology, molecular docking

## Abstract

Atorvastatin is a widely used lipid-lowering drug in the clinic. Research shows that taking long-term atorvastatin has the risk of drug-induced liver injury (DILI) in most patients. Hugan tablets, a commonly used drug for liver disease, can effectively lower transaminase and protect the liver. However, the underlying mechanism of Hugan tablets alleviating atorvastatin-induced DILI remains unclear. To address this problem, comprehensive chemical profiling and network pharmacology methods were used in the study. First, the strategy of “compound−single herb−TCM prescription” was applied to characterize the ingredients of Hugan tablets. Then, active ingredients and potential targets of Hugan tablets in DILI treatment were screened using network pharmacology, molecular docking, and literature research. In the end, the mechanism of Hugan tablets in treating atorvastatin-induced DILI was elucidated. The results showed that Hugan tablets can effectively alleviate DILI induced by atorvastatin in model rats, and 71 compounds were characterized from Hugan tablets. Based on these compounds, 271 potential targets for the treatment of DILI were predicted, and 10 key targets were chosen by characterizing protein–protein interactions. Then, 30 potential active ingredients were screened through the molecular docking with these 10 key targets, and their biological activity was explained based on literature research. Finally, the major 19 active ingredients of Hugan tablets were discovered. In addition, further enrichment analysis of 271 targets indicated that the PI3K-Akt, TNF, HIF-1, Rap1, and FoxO signaling pathways may be the primary pathways regulated by Hugan tablets in treating DILI. This study proved that Hugan tablets could alleviate atorvastatin-induced DILI through multiple components, targets, and pathways.

## Introduction

Hyperlipidemia widely affects the health of the elderly nowadays and is recognized as a social problem (Townsend et al., 2019). In the treatment of hyperlipidemia, atorvastatin is a commonly prescribed lipid-lowering drug (Black, 2002). However, studies showed that the long-term use of atorvastatin could cause serious side effects, represented by liver injury, in which case the level of liver transaminase of the patient would be increased (Clarke and Mills, 2006; Deedwania et al., 2007). Therefore, drug research on decreasing the side effects of statins is of great significance.

In recent years, traditional Chinese medicine (TCM) has made significant breakthroughs in the treatment of drug-induced liver injury (DILI), such as the application of Hugan tablets (Yao et al., 2018). The prescription of Hugan tablets is composed of six medicinal herbs, namely, Bupleuri Radix (BR, derived from *Bupleurum chinense* DC. or *Bupleurum scorzonerifolium* Willd.), Artemisiae Scopariae Herba (ASH, derived from *Artemisia capillaris* Thunb.), Schisandrae chinensis Fructus [SCF, derived from *Schisandra chinensis* (Turcz.) Baill.], Isatidis Radix (IR, derived from *Isatis tinctoria* L.), Pulvis Fellis Suis (PFS, derived from *Sus scrofa domestica* Brisson), and mung bean [MB, derived from *Vigna radiata* (L.) R. Wilczek]. Clinically, Hugan tablets can reduce the level of liver transaminase such as aspartate transaminase (AST), alanine transaminase (ALT), and total bilirubin (TBIL) and is used for chronic hepatitis and early liver cirrhosis (Liu et al., 2019). However, whether Hugan tablets have a therapeutic effect on atorvastatin-induced liver injury remains unclear, and its mechanism of action has not yet been elucidated.

Network pharmacology has been successfully applied to the construction of networks that characterize the relationship between drugs, targets, and diseases, and it has played an active role in the research of TCM prescriptions due to its integrity and systematicness (Hopkins, 2008). However, a major challenge restricting its application is the accurate characterization of active ingredients. At present, most of the network pharmacology studies are based on the components reported in the database yet do not experimentally investigate the content of ingredients, which is closely related to the medicinal effects. Therefore, in this study, the strategy of “compound–single herb–TCM prescription” based on ultrahigh-performance liquid chromatography coupled with a quadrupole time-of-flight tandem mass spectrometry (UPLC-Q-TOF-MS/MS) (Xing et al., 2017) was applied to characterize the ingredients of Hugan tablets, which provides a solid material foundation for the follow-up network pharmacology and molecular docking.

**FIGURE 1:**
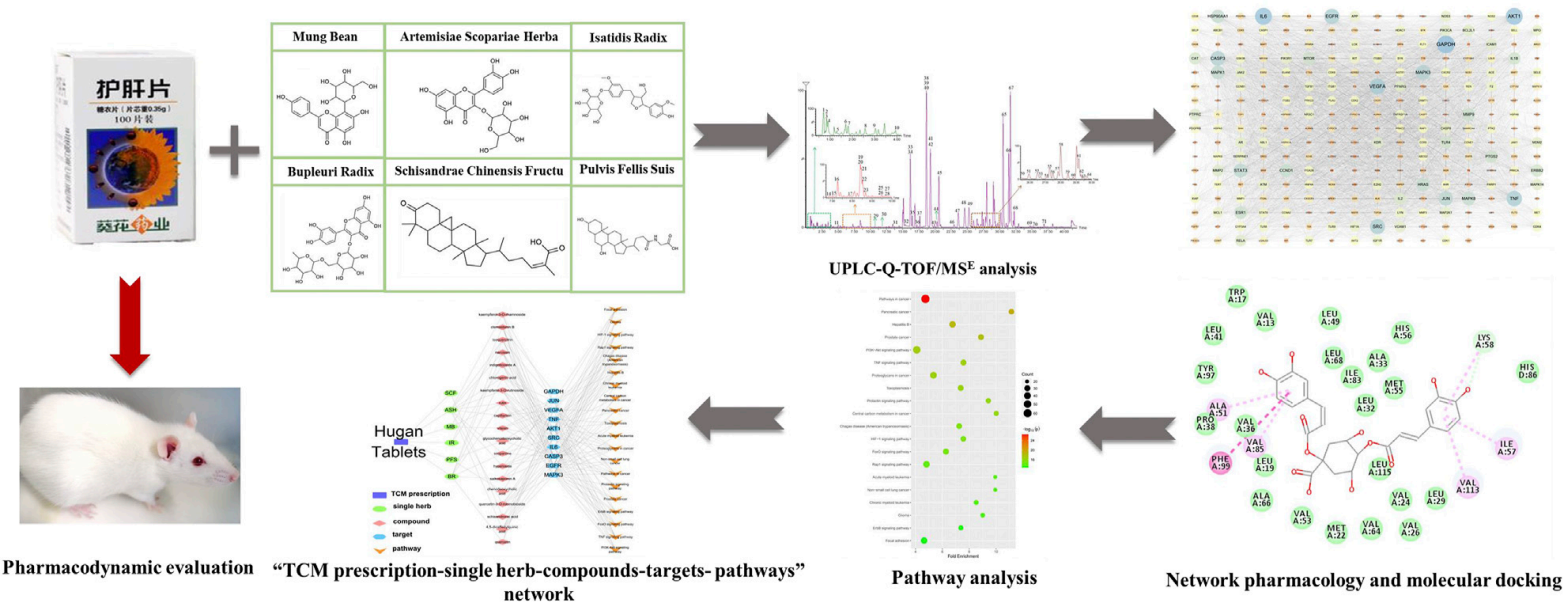
Flowchart for the investigation of the mechanism of Hugan tablets in treating drug-induced liver injury induced by atorvastatin.

In this study, the effect of Hugan tablets on the treatment of DILI was validated in rat models and then the ingredients of Hugan tablets were characterized. Based on this material basis, the major active ingredients and targets of Hugan tablets were selected by network pharmacology, molecular docking, and literature research. At last, the potential mechanism of Hugan tablets was analyzed (Figure 1). This study demonstrated the huge potential of TCM for ameliorating the side effects of chemical drugs.

## Materials and Methods

### Chemicals and Reagents

Atorvastatin calcium tablets (batch No. 170518) were obtained from Jialin Pharmaceutical (Beijing, China). AST, ALT, TBIL, and alkaline phosphatase (ALP) assay kits were obtained from Beckman Kurt Experimental System Co., Ltd. (Suzhou, China). Hugan tablets (batch No. 201710078), BR, ASH, SCF, IR, PFS, and MB were obtained from Sunflower Pharmaceutical Group Co., Ltd. (Harbin, China).

Mass spectrometry–grade acetonitrile, methanol, and formic acid were obtained from Fisher Scientific. The reference standards of quercetin, rutin, scopoletin, hyperoside, scoparone, chlorogenic acid, saikosaponin B2, schisantherin A, schisandrin A, schisandrin B, schisandrin C, schisandrol A, schisandrol B, and schisanhenol were obtained from Chengdu Purifa Technology Development Co., Ltd. (Chengdu, China). Chenodeoxycholic acid (CDCA) was obtained from Shanghai Yuanye Biotechnology Co., Ltd. (Shanghai, China). Vitexin was obtained from Shanghai Aladdin Biochemical Technology Co., Ltd. (Shanghai, China). Saikosaponin A, proline, and hyodeoxycholic acid (HDCA) were obtained from the National Institutes for Food and Drug Control (Beijing, China). Arginine was obtained from Shanghai Macklin Biochemical Co., Ltd. (Shanghai, China). The purity of all standards was over 98%, as determined by HPLC analysis.

### Animals

Male Sprague Dawley (SD) rats weighing 190–210 g were raised in the Animal Experimental Center of Beijing University of Chinese Medicine. Animal experiments were approved by the Animal Care and Ethics Committee of Beijing University of Chinese Medicine (approval number: BUCM-4-2018091304–3,023). The environmental temperature was controlled at 21–25°C with a relative humidity of 55–65% (12 h/12 h day/night cycle), and the pads were changed four times a week. Rats were allowed to adapt to the environment for 7 days with free food and water before the experiment.

### Pharmacological Experiment Design and Preparation of Bio-Samples

For the dosage of Hugan tablets, according to the clinical dosage, 0.504 g kg^−1^·d^−1^ was set as the low dosage, and the double-dose 1.008 g kg^−1^·d^−1^ was set as the high dosage. The dosage of atorvastatin calcium tablets suspension was 50 mg kg^−1^.

At the beginning, 68 SD rats were randomly assigned into three groups: 34 in the atorvastatin group, 17 in the control group, and 17 in the prevention group. The atorvastatin group received the intragastric administration of atorvastatin calcium tablet suspension. The prevention group received the intragastric administration of atorvastatin calcium tablet suspension plus high-dose Hugan tablets at the same time. The control group was given water of the same volume. After 50 days, the rats in the initial atorvastatin group were identified as successful models and were subsequently divided into two groups randomly, with some rats remaining within the atorvastatin group and the others moved to the treatment group. The treatment group received the intragastric administration of high-dose Hugan tablets, instead of atorvastatin calcium tablet suspension. Finally, there were four groups of experimental animals. The continuous administration was conducted for 14 days. After the last administration, all rats were fasted for 12 h and given water freely. Then, once the rat was anesthetized with chloral hydrate, blood was drawn from the main abdominal vein and the liver was peeled off as soon as possible to evaluate the efficacy of the Hugan tablets.

The blood samples were centrifuged at 3,000 rpm for 15 min to separate serum for biochemical analysis. Serum ALT, AST, ALP, and TBIL levels were measured with a CX4 Pro automatic biochemical analyzer (Beckman Coulter Inc., United States). The left lateral lobe of the liver was fixed in 10% formalin, then embedded in paraffin, sectioned, and stained with hematoxylin–eosin (HE). An optical microscope was used to observe the histological sections.

### Statistical Analysis

SPSS software 17.0 (SPSS Inc., Chicago, United States) was used for statistical analysis. All data are presented as mean ± SD. Differences between multiple groups were examined using the one-way ANOVA. The LSD-t test was adopted to compare the data between two groups. The significance levels were *p < 0.05, **p < 0.01.

### Preparation of Sample and Reference Solution for LC-MS/MS

Hugan tablets composed of 4.2 g BR, 4.2 g ASH, 4.2 g IR, 2.2 g SCF, 0.3 g PFS, and 0.2 g MB. First, Hugan tablets, BR, ASH, SCF, IR, PFS, and MB were crushed. Then, 2.0 g of powder was accurately weighed and transferred to a 50-ml triangular flask with plug, in which 25 ml of 70% methanol was added. After 1 h ultrasound-assisted extraction, the extract solution was centrifuged at 12,000 r/min for 10 min and then filtered through a 0.22-μm filter membrane before qualitative analysis. Every single standard solution was prepared by dissolving accurately weighed standards in methanol and stored at 4°C in the dark.

### UPLC-Q-TOF-MS/MS Analysis

The analysis was performed on a Waters ACQUITY UPLC I-Class system coupled with the Waters SYNAPT G2-Si Mass Spectrometer *via* an electrospray ionization (ESI) interface. Chromatographic separation was performed using an ACQUITY UPLC BEH C18 column (2.1 × 100 mm, 1.7 μm) held at 40°C, and the flow rate was 0.3 ml/min. The mobile phases consisted of 0.1% formic acid aqueous solution (A) and acetonitrile (B), using a linear gradient program as follows: 0–9 min, 2–20% B; 9–12 min, 20–30% B; 12–17 min, 30–40% B; 17–24 min, 40–42% B; 24–38 min, 42–98% B; and 38–40 min, 98% B.

The optimal mass spectrometer parameters were employed as follows: capillary voltage, 3 kV; cone voltage, 40 V; source temperature, 100°C; desolvation gas temperature, 300°C; and desolvation gas flow, 600 L/h. MS measurement was obtained in the MS^E^ mode, and the collision energy of low energy function and high energy function was set at 6 V and 20–40 V, respectively. The acquisition mass range was 50–1,200 Da in both negative and positive ion modes. Data were acquired and analyzed by Waters MassLynx V4.1 and Waters UNIFI 1.71 software.

### Identification of Compounds in Hugan Tablets

The identification procedure was mainly divided into five steps (Wang et al., 2016; Wang et al., 2017). 1) The mass spectrometry data were collected using the aforementioned methods. 2) A database of chemical compounds of each medicinal herb in Hugan tablets (including the name, molecular formula, and structural formula of each compound) was built by the SciFinder database (https://www.cas.org/products/scifinder), the ChemSpider database (http://www.chemspider.com/), and relevant literature reports. 3) The peaks were screened preliminarily by the UNIFI^TM^ platform based on the in-house database and the self-built database. The known compounds were validated further based on the accurate mass, fragment ions, neutral losses, retention behaviors, reference standards, and previous reports. 4) For the potential novel compounds, based on the accurate mass, fragment ions, characteristic fragments, and retention behaviors, possible structures were obtained by combining UniFi’s Elucidate function with the literature, ChemSpider, Mass Bank (https://massbank.eu/MassBank/), and other databases, and then entered them into UniFi software for further verification. 5) The compounds in single herb were identified and then the ingredients in the TCM prescription were characterized by comparing the peaks in prescription with the corresponding peaks in each single herb.

### Target Collection

The potential targets of the compounds in Hugan tablets were searched from SwissTargetPrediction (http://www.swisstargetprediction.ch/) (Gfeller et al., 2014) and TCMSP (Ru et al., 2014). The biological targets related to DILI were selected from the Online Mendelian Inheritance in Man (OMIM, http://www.omim.org/) and the GeneCards database (https://www.genecards.org/). The protein names of these targets were converted into their official gene names via UniProtKB (http://www.uniprot.org/). Then, the Venn diagram was drawn to obtain the intersected drug-related targets and the disease-related targets, which are potential targets for Hugan tablets to treat DILI.

### Pathway Enrichment of Potential Targets

The Database for Annotation, Visualization, and Integrated Discovery (DAVID, http://david.abcc.ncifcrf.gov/home.jsp, version 6.8) was employed to analyze the KEGG pathways of the predicted targets. The R package ggplot2 was used to create the bubble plot.

### Protein–Protein Interaction Network Construction

All potential targets were uploaded into the STRING database (http://string-db.org/) (Szklarczyk et al., 2017) to analyze their interactions. The PPI data were imported into Cytoscape v3.7.1 (http://cytoscape.org/) to construct a PPI network and perform topological analysis. The top 10 targets ranked by degree were selected to screen the potential active ingredients of Hugan tablets based on molecular docking.

### Screening of Active Ingredients of Hugan Tablets

The crystal structures of the ten key proteins were downloaded from the RCSB (http://www.rcsb.org/) database. Discovery Studio 4.0 (DS) LibDock was used to molecularly dock the compounds of Hugan tablets with the ten target proteins. For the target protein with the original co-crystal ligand available, the cutoff value was the LibDock score of the protein and its corresponding original ligand (Rao et al., 2007). For the protein without original co-crystal ligand, the cutoff value was the LibDock score of the protein and its corresponding approved positive drug (Hu et al., 2012). Compounds with a higher LibDock score than the cutoff value were considered potential active ingredients of Hugan tablets. Then, literature investigation into the potential active ingredients was carried out to obtain information on the hepatoprotective effects of these ingredients and their contents in a single herb, so as to eliminate some false-positive results of virtual screening and finally screen the major active ingredients of Hugan tablets.

### “TCM Prescription-Single Herb-Compounds-Targets-Pathways” Network Construction

The potential active ingredients, key target proteins, and corresponding top 20 pathways obtained by the previous screening were imported into Cytoscape 3.6.1 software, and the “Merge” plug-in in Cytoscape software was used to construct the “TCM prescription–single herb–compounds–targets–pathways” network of Hugan tablets in the treatment of DILI.

## Results

### Evaluation of the Preventive and Therapeutic Effects of Hugan tablets

**FIGURE 2:**
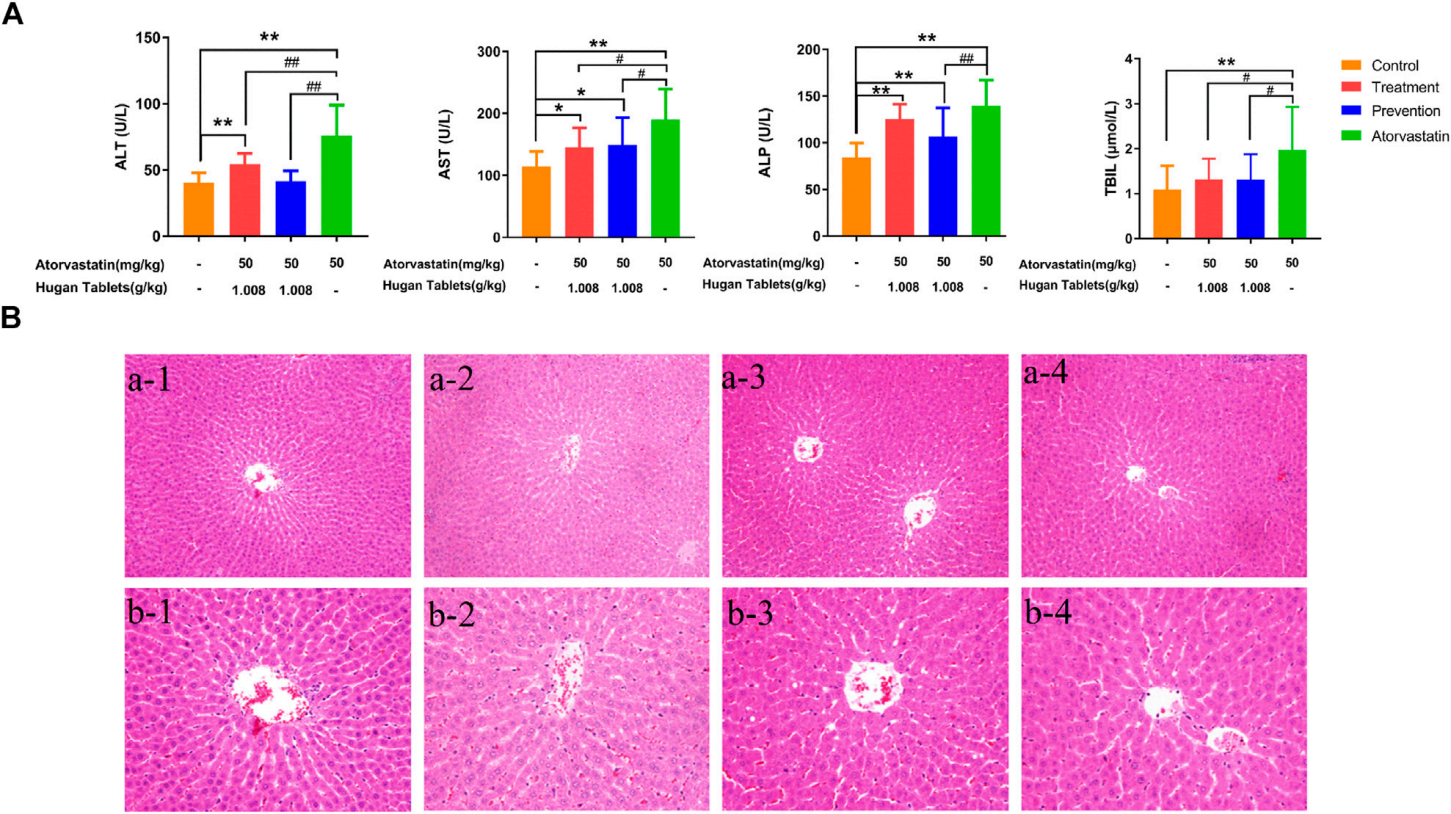
Efficacy of Hugan tablets in ameliorating DILI. **(A)** Effects of Hugan tablets on alleviating elevated serum ALT, AST, ALP, and TBIL, resulting from atorvastatin-induced liver injury (n_Control_ = 13; n_Treatment_ = 10; n_Prevention_ = 15; n_Atorvastatin_ = 12). Data are presented as mean ± SD. ^*^ *p* < 0.05, **p < 0.01 vs. control group, ^#^ *p* < 0.05, ^##^ *p* < 0.01 vs. atorvastatin group. **(B)** Histomorphology of liver tissues of rats in different groups (HE, a × 200, b × 400). 1: control group; 2: atorvastatin group; 3: prevention group; 4: treatment group.

The serum levels of ALT, AST, ALP, and TBIL were the biochemical factors most commonly used to evaluate the liver function (Boone et al., 2005; Kwo et al., 2017; Lei et al., 2020). Compared with the control group, the serum levels of ALT, AST, ALP, and TBIL of the atorvastatin group were significantly increased (*p* < 0.01) (Figure 2A), which indicated that the model was successfully established. Before dosing of atorvastatin, the preventive administration of Hugan tablets could significantly reduce the levels of ALT, TBIL, AST, and ALP in rats with DILI. The amelioration effect was further observed as levels of ALT and TBIL, which showed no significant difference from the control group, while AST and ALP levels are still significantly higher than those of normal rats (*p* < 0.05; *p* < 0.01). After dosing of atorvastatin, the therapeutic administration of Hugan tablets significantly reduced the ALT, AST, and TBIL levels of the DILI model rats and made the TBIL level comparable to normal rats, while the levels of ALT and AST did not reach the normal level. Taken together, the results showed that Hugan tablets had significant ameliorating effects on DILI. However, in the results of HE staining, no significant alteration in the liver tissues was observed among the control group, atorvastatin group, prevention group, and treatment group, indicating that the degree of injury is light, and it has not yet been reflected in the pathology (Figure 2B).

### Identification of Compounds in Hugan Tablets

**FIGURE 3:**
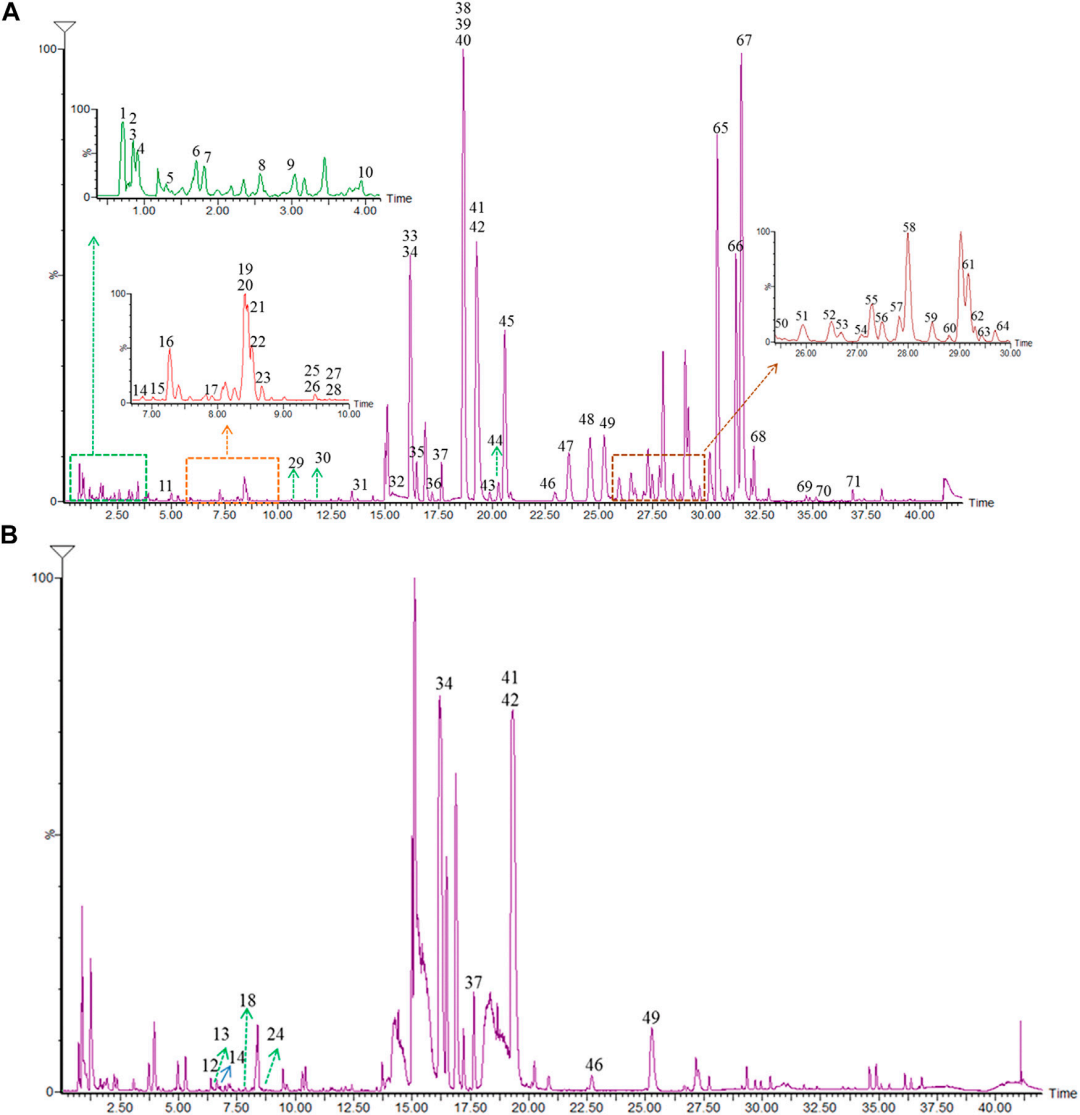
BPC of Hugan tablets in both positive **(A)** and negative **(B)** ion modes.

To investigate the underlying mechanisms of Hugan tablets in the DILI induced by atorvastatin, the ingredients of Hugan tablets were clarified. A problem for the analysis of TCM prescriptions was that if the compounds were identified directly, the peak with a high response might depress the small peak, thus increase the difficulty of identification. Therefore, the strategy of “compounds-single herb-TCM prescription,” which was successfully developed in our previous research, was used to characterize the ingredients of Hugan tablets (Wang et al., 2017). First, the reference substances of various analogies in each herb were analyzed by high-resolution mass spectrometry (HRMS), and the diagnostic ions and fragmentation rules were summarized. By using data post-processing methods such as extracting diagnostic ions, comparing fragment pathways, and filtering neutral loss, the components in each single herb were identified. Then, the ingredients in the TCM prescription were characterized by comparing the peaks in the prescription with those identified in each single herb. The base peak chromatograms (BPCs) of Hugan tablets in both positive and negative ion modes are presented in Figure 3. In addition, the BPC of Hugan tablets and its medicinal herb is shown in Supplementary Figure S1.

**FIGURE 4:**
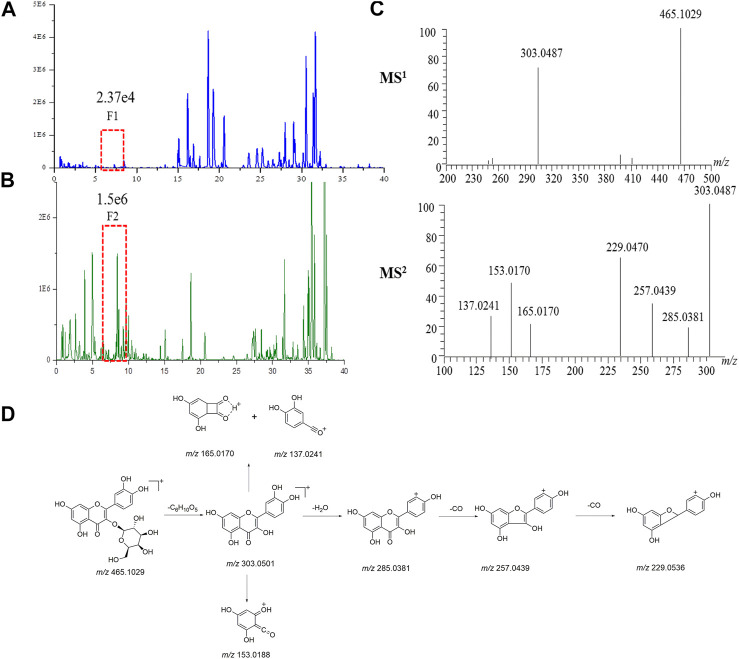
Identification of compounds in Hugan tablets. Chromatograms of common components in Hugan tablets **(A)** and BR **(B)** in positive ion mode **(C)** The MS^1^ spectrum and MS^2^ spectrum of F2. **(D)** The fragmentation pathway of hyperoside.

Take the identification of a compound in ASH as an example, in the BPC of ASH (Figure 4B), the F2 peak (1.56e^6^) with a higher response was observed, while at the same retention time, the F1 peak response in the BPC of Hugan tablets (Figure 4A) was lower (2.37e^4^). Therefore, first, F2 can be identified to infer F1 due to the same retention time. F2 provided the precursor ion of [M + H]^+^ at *m/z* 465.1029, as well as fragment ions at *m/z* 303.0487 [M + H-C_6_H_10_O_5_]^+^, *m/z* 153.0182 [M + H-C_14_H_16_O_8_]^+^, and *m/z* 137.0241 [M + H-C_14_H_16_O_9_]^+^, which were characteristic fragment ions of flavonols (Figure 4C). Thus, F2 was confirmed as hyperoside by comparing with the MS^n^ data and retention time of the reference standard. The possible cleavage pathway of hyperoside is shown in Figure 4D. This method can not only effectively identify compounds with low content but also increase the reliability of compound identification in the TCM prescription.

We have comprehensively characterized 71 compounds of Hugan tablets, including 11 flavonoids and their glycosides, 8 triterpene saponins, 3 coumarins, 29 lignans, 8 organic acids, 5 bile acids, 3 nucleic acids, and 4 other compounds. The retention time and the MS^n^ data of the characterized compounds are summarized in Supplementary Table S1.

### Potential Targets of Hugan Tablets in Treating Drug-Induced Liver Injury

**FIGURE 5:**
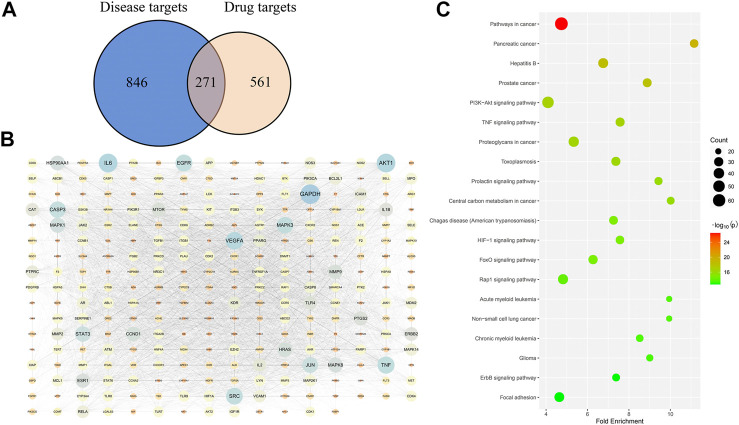
Network pharmacology–based analysis of the treatment of DILI with Hugan tablets. **(A)** Common targets of Hugan tablets and DILI. **(B)** PPI network of potential targets. **(C)** KEGG pathway enrichment results.

Among the 71 compounds, 832 drug targets were searched from the SwissTargetPrediction and TCMSP databases. A total of 1,117 disease targets of DILI were obtained from the OMIM and Genecards databases. Then, the Venn diagram was drawn to obtain 271 overlapped targets (Figure 5A), which are potential targets for Hugan tablets in treating DILI. For specific target information, see Supplementary Table S2.

### Protein–Protein Interaction Network Analysis

The 271 potential targets were introduced into STRING to obtain the information on predicted interaction, and Cytoscape software was used to construct and analyze the PPI network (Figure 5B). Network topology analysis was performed to get the degree value of these targets. The targets with a higher degree may represent the key targets for the treatment of DILI by Hugan tablets. Thus, the top ten targets ranked by degree value were collected, namely, GAPDH, IL6, AKT1, VEGFA, TNF, EGFR, SRC, MAPK3, CASP3, and JUN (Table 1).

**TABLE 1:** Ten key targets of Hugan tablets in treating DILI.

NO.	Gene symbol	Uniprot Id	Description	Degree	Pdb
1	GAPDH	P04406	Glyceraldehyde-3-phosphate dehydrogenase	183	1U8F
2	IL6	P05231	Interleukin-6	172	1ALU
3	AKT1	P31749	RAC-alpha serine/threonine-protein kinase	172	6HHF
4	VEGFA	P15692	Vascular endothelial growth factor A	155	4QAF
5	TNF	P01375	Tumor necrosis factor	149	7KP9
6	EGFR	P00533	Epidermal growth factor receptor	146	3POZ
7	SRC	P12931	Proto-oncogene tyrosine-protein kinase src	144	2H8H
8	MAPK3	P27361	Mitogen-activated protein kinase 3	140	4QTB
9	CASP3	P42574	Caspase-3	139	1GFW
10	JUN	P05412	Transcription factor AP-1	130	5FV8

### Active Ingredients of Hugan Tablets

The molecular docking score can reflect the affinity of the compound and the target, which can be used to virtually screen the compounds more likely to interact with potential targets. Based on molecular docking, 30 compounds with a higher LibDock score than the cutoff value were considered as potential active ingredients of Hugan tablets (Supplementary Table S3). Detailed information of the docking scores and cutoff values are presented in Supplementary Table S4.

Next, we conducted literature research on the biological activities of 30 potential active ingredients and their content in a single herb, and excluded the ingredients with little content or whose biological activity related to liver protection has not been verified. Finally, 19 active ingredients of Hugan tablets were screened out (Table 2), including 10 flavonoids, 5 phenylpropanoids, 1 triterpenoid saponin, 2 and 1 tetracyclic triterpenoid. It was found that the active ingredients in Hugan tablets mainly exhibit antioxidant, anti-inflammatory, and liver fibrosis–inhibiting effects, to play a role in protecting the liver. It is worth noting that most of the 10 flavonoids belong to flavonols, suggesting that the introduction of the 3-hydroxyl group in the flavonoid core structure may be important for their liver-protecting activity.

**TABLE 2:** Screening results of active ingredients of Hugan tablets.

Type of compounds	Compounds	CAS	Molecular formula	Structural formula	Herb source	Biological activity	References
Flavonoids	Hyperoside	482–36–0	C_21_H_20_O_12_	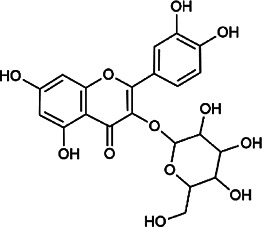	BR, ASH	It suppresses hepatic oxidative stress by activating endogenous antioxidant mechanisms and protects against liver fibrosis	Niu et al. (2017), Guo et al. (2019)
	Kaempferol-3-*O*-rutinoside	17,650–84–9	C_27_H_30_O_15_	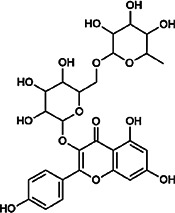	ASH	It probably mediates a strong hepatoprotective effect *via* the reduction of oxidative stress and apoptotic cell death	Wang et al. (2015)
	Quercetin-3-*O*-robinobioside	52,525–35–6	C_27_H_30_O_16_	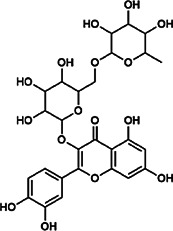	ASH	It has high antioxidant activity	Zhu et al. (2020)
	Rutin	153–18–4	C_27_H_30_O_16_	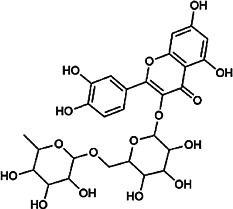	BR, MB, ASH	It ameliorates hepatic injury *via* the IL-6/STAT3 pathway, and interfering with oxidative stress, inflammation, and apoptosis	Hafez et al. (2015), Caglayan et al. (2019)
	Narcissin	604–80–8	C_28_H_32_O_16_	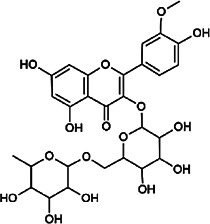	BR, ASH	It reveals significant antioxidant and hepatoprotective effects	Gevrenova et al. (2015)
	Kaempferol-3-*O*-rhamnoside	482–39–3	C_21_H_20_O_10_	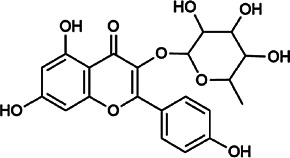	ASH	It has a hepatoprotective effect on the lipopolysaccharide/d-galactosamine–induced acute liver failure	Hu et al. (2018)
	Isoquercitrin	21,637–25–2	C_21_H_20_O_12_	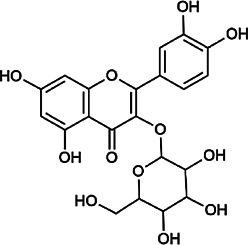	BR, ASH, MB	It protects the liver though inhibition of oxidative stress, nitrosative stress, and inflammation	Xie et al. (2016)
	Vitexin	3,681–93–4	C_21_H_20_O_10_	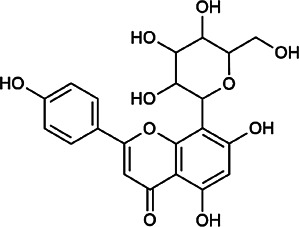	MB	It ameliorates the liver disease by inhibiting inflammation	Li et al. (2020)
	Capillarisin	56,365–38–9	C_16_H_12_O_7_	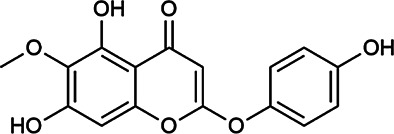	ASH	It suppresses oxidative stress and prevents liver apoptosis primary hepatocytes *via* the reduction of HO-1 expression and inactivation of its downstream target NF-κB	Lee et al. (2009)
	Quercetin	117–39–5	C_15_H_10_O_7_	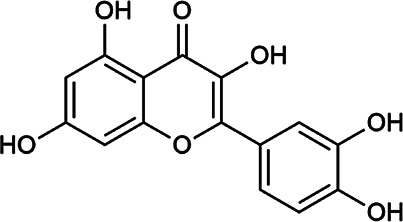	BR	It significantly minimizes oxidative stress as well as inflammatory response in hepatic cells	Lee et al. (2019)
Phenylpropanoids	Indigoticoside A	143,663–00–7	C_26_H_34_O_11_	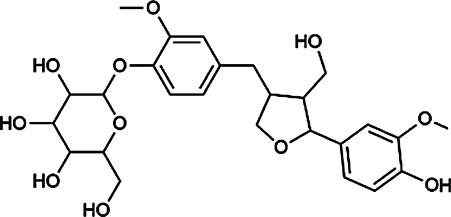	RI	It has a strong antioxidant activity	Xiao et al. (2014)
	Clemastanin B	112,747–98–5	C_32_H_44_O_16_	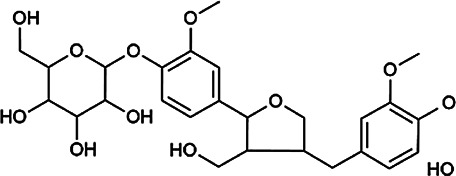	IR	It has a strong antioxidant activity	Xiao et al. (2014)
	Scoparone	120–08–1	C_11_H_10_O_4_	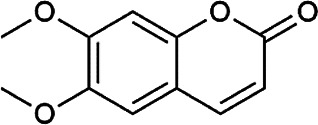	BR, ASH	It attenuates hepatic stellate cell activation through inhibiting TGF-β/Smad signaling pathway	Liu and Zhao, (2017)
	4,5-Dicaffeoylquinic acid	57,378–72–0	C_25_H_24_O_12_	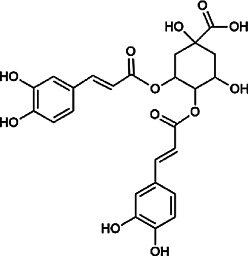	ASH	It is the anti-inflammatory and anti-HBV components in ASH.	Tan et al. (2008); Zhao et al. (2014)
	Chlorogenic acid	327–97–9	C_16_H_18_O_9_	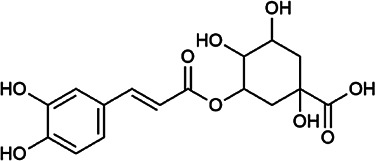	BR, ASH	It inhibits liver injury and might be related with antioxidant and anti-inflammatory	Shi et al. (2016)
Triterpenoid saponins	Saikosaponin A	20,736–09–8	C_42_H_68_O_13_	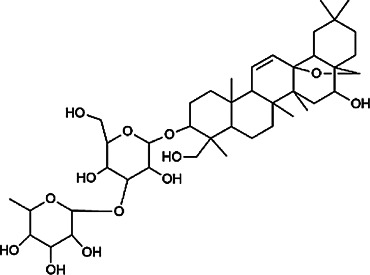	BR	It downregulates BMP-4 expression and inhibits hepatic stellate cell activation to inhibit liver fibrosis; it inhibits the expression of hepatic pro-inflammatory cytokines and the NF-κB signal pathway and increases the expression of anti-inflammatory cytokine IL-10, thereby inhibiting liver injury	Wang et al. (2013), Wu et al. (2008)
Bile acids	Glycochenodeoxycholic acid	640–79–9	C_26_H_43_NO_5_	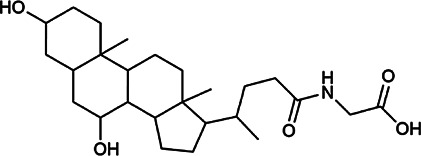	PFS	It shows a strong association with fibrosis	Kwan et al. (2020)
	Chenodeoxycholic acid	474–25–9	C_24_H_40_O_4_	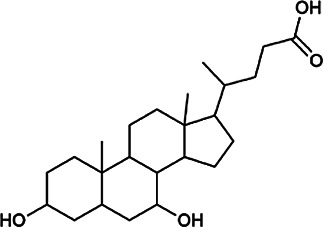	PFS	It ameliorates liver fibrosis by inhibiting TIMP-1 expression resulting from activation of farnesoid X receptor	Kang et al. (2012)
Tetracyclic triterpenoids	Schisandronic acid	55,511–14–3	C_30_H_46_O_3_	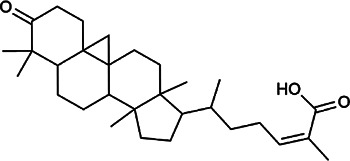	SCF	It has an antioxidant activity	Tasneem et al. (2021)

### Pathway Analysis

Based on the DAVID database, a pathway enrichment analysis of targets was performed to identify the potential pathways intervened by Hugan tablets administration. A total of 139 potential pathways were obtained, with a cutoff *p*-value of 0.05, and the top 20 pathways (with the lowest *p*-values) are shown in Figure 5C. The number of targets contained in each pathway suggests that Hugan tablets mainly exert its protective effect on DILI by regulating the PI3K-Akt, TNF, HIF-1, Rap1, and FoxO signaling pathways.

### “TCM Prescription–Single Herb–Compounds–Targets–Pathways” Network Construction

**FIGURE 6:**
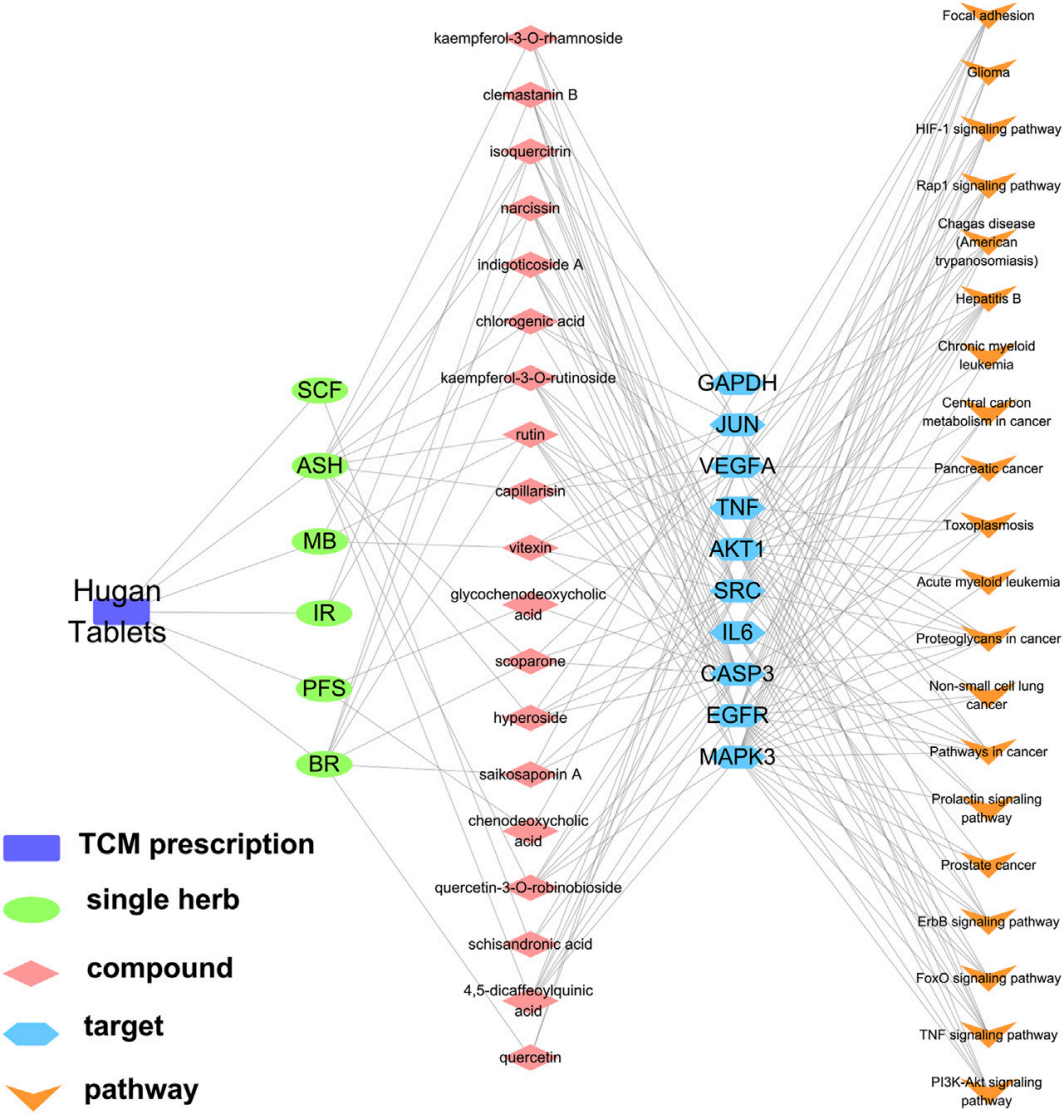
Network diagram of “TCM prescription–single herb–compounds–targets–pathways.”

According to the 19 potential active ingredients, 10 key targets, and corresponding top 20 pathways screened earlier, the “TCM prescription–single herb–compounds–targets–pathways” network of Hugan tablets in the treatment of DILI was constructed (Figure 6). In this network, the target(s) and the pathway(s) that the compound(s) in Hugan tablets act on could be summarized comprehensively. For example, compounds such as clemastanin B, rutin, 4,5-dicaffeoylquinic acid, and chlorogenic acid may act on the PI3K-Akt signaling pathway by binding IL6, AKT1, VEGFA, or MAPK3.

Besides, it could be found that drug–target interactions exist not only in the mode of “single target binding to multiple compounds” but also in the mode of “single compound binding to multiple targets”. For example, MAPK3 can bind to clemastanin B, kaempferol-3-*O*-rutinoside, quercetin-3-*O*-robinobioside, rutin, and 4,5-dicaffeoylquinic acid, while rutin acts on AKT1, EGFR, SRC, MAPK3, and CASP3, which were involved in multiple pathways. This indicated that Hugan tablets influenced DILI by comprehensive regulation of multiple compounds, targets, and pathways.

## Discussion

The research on the mechanism of TCM has always been challenging because it contains multiple components, targets, and pathways. Network pharmacology explores the relationship between drugs and diseases from a global perspective by constructing networks and is an ideal tool for studying the pharmacology of TCM for the treatment of diseases.

In order to provide a solid pharmacodynamic prerequisite and material basis for network pharmacology, we verified the efficacy of Hugan tablets in a model of atorvastatin-induced liver injury and then identified 71 compounds from Hugan tablets. Based on network pharmacology, molecular docking, and literature research, 19 active ingredients, 10 key targets, and some important pathways of Hugan tablets anti-DILI were obtained, and a “TCM prescription–single herb–compounds–targets–pathways” network was constructed.

Liver injury is usually accompanied by inflammatory responses. Inflammatory factors such as transforming growth factor-beta (TGF-β) and tumor necrosis factor (TNF) are key inflammatory regulators in the progress of the liver disease, which can cause or aggravate liver cell damage (Jaeschke, 2006; Kubes and Jenne, 2018). Therefore, inhibition of inflammation is of great significance for the prevention and treatment of liver injury. In the acute tissue injury stage, the dysregulated TNF-α signaling can activate an inflammatory cascade to trigger excessive release of cytokines/chemokines and cell death (Lai et al., 2019). Therefore, Hugan tablets may regulate TNF signaling pathway through IL-6, AKT1, TNF, MAPK, CASP3, and JUN, thereby inhibiting inflammation and protecting the liver cells from apoptosis. It has been reported that the knockout of repressor and activator protein (Rap1) reduced the hepatic damage and hepatic inflammatory response (Li et al., 2016). So, it is speculated that Hugan tablets may inhibit the Rap1 signaling pathway by acting on AKT1, VEGFA, EGFR, SRC, and MAPK3, thereby preventing inflammation and protecting the liver.

Oxidative stress is closely related to almost all human liver diseases. The FoxO signaling pathway is involved in cell apoptosis induced by oxidative stress, and studies have shown that FoxO3 has a pro-apoptotic effect on liver cell under oxidative stress (Tao et al., 2013). Therefore, Hugan tablets may regulate the FoxO3 signaling pathway by acting on the targets of IL6, AKT1, EGFR, and MAPK3 to alleviate the apoptosis of the liver cells under oxidative stress.

**FIGURE 7:**
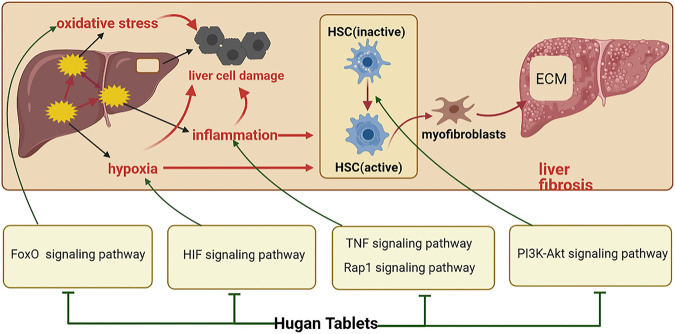
Potential mechanism of Hugan tablets in treating DILI.

In addition, liver injury and inflammation cause the activation of the liver tissue immune system for tissue repair. When the repair is excessive and out of control, hepatic stellate cells (HSCs) are activated (Puche et al., 2013; Higashi et al., 2017), and the extracellular matrix (ECM) secreted by them is excessively deposited in the liver, resulting in abnormal liver structure and liver function. This process can also be called liver fibrosis (Jiang et al., 2017). Studies have shown that the activation of the PI3K-Akt signaling pathway can promote the proliferation of HSC, thereby accelerating the occurrence and development of liver fibrosis (Zhang et al., 2019; Du et al., 2020). Therefore, Hugan tablets may inhibit the activation of the PI3K-Akt signaling pathway through IL-6, AKT1, VEGFA, EGFR, and MAPK3, thereby inhibiting the activation of HSCs and delaying the occurrence of liver fibrosis. In the early stage of the disease, liver injury can also cause local tissue hypoxia, which can aggravate the cell damage and inflammation and promote liver fibrosis. These effects are mediated by hypoxia-inducible factor (HIF) (Rosmorduc and Housset, 2010), so Hugan tablets may regulate the HIF signaling pathway by regulating the expression of GAPDH, IL-6, AKT1, VEGFA, and MAPK3, so as to alleviate the adverse effects of hypoxia to protect the liver. In summary, Hugan tablets may alleviate DILI by resisting oxidative stress and inhibiting inflammation and hepatic fibrosis (Figure 7). However, this study still has limitations, for example, the key targets and pathways of Hugan tablets need to be verified by *in vivo* and *in vitro* experiments.

## Conclusion

In this study, it was found that Hugan tablets can effectively alleviate atorvastatin-induced DILI. Seventy-one compounds in Hugan tablets were characterized, which provided an important basis for elucidating the substance basis of Hugan tablets. Network pharmacological analysis results indicated that Hugan tablets could inhibit inflammatory and alleviate hepatic fibrosis through the comprehensive regulation of multiple compounds, targets, and pathways, thereby achieving an anti-DILI effect.

## Data Availability

The original contributions presented in the study are included in the article/Supplementary Material, further inquiries can be directed to the corresponding author.

## Abbreviations

ASH, Artemisiae Scopariae Herb; BR, Bupleuri Radix; DILI, drug-induced liver injury; IR, Isatidis Radix; MB, mung bean; PFS, Pulvis Fellis Suis; SCF, Schisandrae Chinensis Fructus.
