# Supplementary material for: The Study on the Mechanism of Hugan Tablets in Treating Drug-Induced Liver Injury Induced by Atorvastatin
Source: Front Pharmacol. 2021 Jun 28;12:683707. doi: 10.3389/fphar.2021.683707 (PMC8275032; doi:10.3389/fphar.2021.683707)
Supplement: Supplementary file 1 [file DataSheet1.doc]

***Supplementary Material***

**Supplementary Figures and Tables**

**Supplementary Figures**


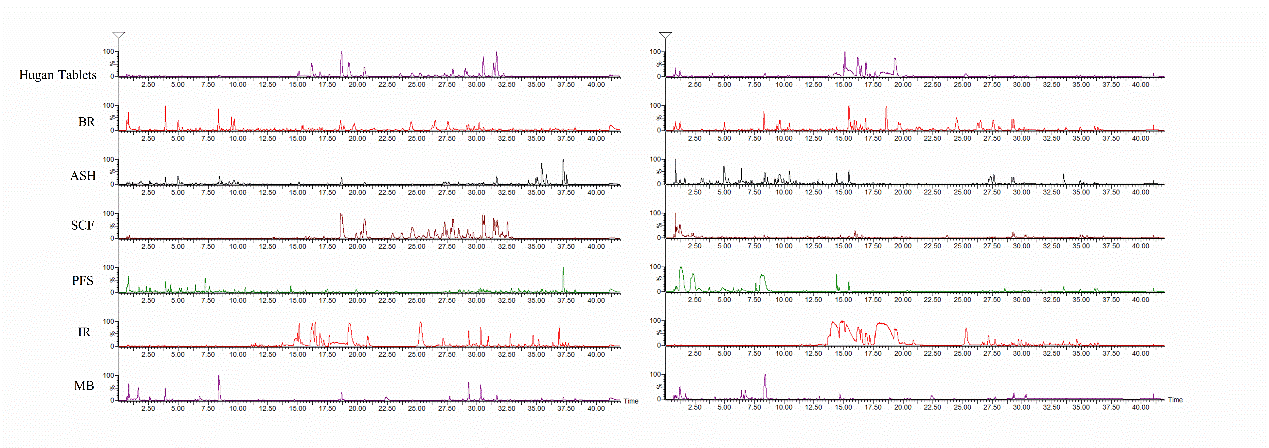


**Supplementary Figure 1.** Chemical characterization of Hugan Tablets and its six medicinal herbs. Left: in positive ion mode; Right: in negative ion mode.

**Supplementary Tables**

**Supplementary Table 2.** Compounds identified in Hugan Tablets by UPLC/Q-TOF-MS/MS.

| **NO** | **Identification** | **Observed （m/z）** | **Adducts** | **Mass error (ppm)** | **t_R_ (min)** | **Response** | **Formula** | **MS/MS** | **Herb Source** |
| --- | --- | --- | --- | --- | --- | --- | --- | --- | --- |
| 1 | Arginine * | 175.1187 | [M+H]^+^ | -1.5 | 0.81 | 27471 | C_6_H_14_N_4_O_2_ | 175.1189[M+H]^+^  158.0922[M+H-NH_3_]^+^  157.1078[M+H-OH]^+^  112.0863[M+H-HCOOH-NH_3_]^+^ | IR，BR |
| 2 | Valine | 118.0859 | [M+H]^+^ | -3.3 | 0.85 | 20492 | C_5_H_11_NO_2_ | 118.0858[M+H]^+^ 72.0809[M+H-HCOOH]^+^ | IR |
| 3 | Proline* | 116.0704 | [M+H]^+^ | -1.7 | 0.85 | 14589 | C_5_H_9_NO_2_ | 116.0705[M+H]^+^ 70.0652[M+H-HCOOH]^+^ | BR, ASH |
| 4 | Adenine | 136.062 | [M+H]^+^ | 1.9 | 0.92 | 23768 | C_5_H_5_N_5_ | 136.0620[M+H]^+^ 119.0350[M+H-NH3]+ | IR, BR |
| 5 | p-Coumaric acid | 165.0549 | [M+H]^+^ | 1.8 | 1.44 | 1160 | C_9_H_8_O_3_ | 165.0546[M+H]^+^ 131.0460[M+H-2OH]^+^ 119.0488[M+H-CH_2_O_2_]^+^ | BR |
| 6 | Adenosine | 268.1035 | [M+H]^+^ | -1.8 | 1.71 | 89095 | C_10_H_13_N_5_O_4_ | 268.1035[M+H]^+^ 136.0618[M+H-C5H8O4]^+^ 119.0352[M+H-C5H8O4-NH3]^+^ | IR |
| 7 | Guanine | 152.0567 | [M+H]^+^ | 0.2 | 1.81 | 71069 | C_5_H_5_N_5_O | 152.0567[M+H]^+^ 135.0300[M+H-NH_3_]^+^ | IR |

Table S1 (Continued)

| **NO** | **Identification** | **Observed （m/z）** | **Adducts** | **Mass error (ppm)** | **t_R_ (min)** | **Response** | **Formula** | **MS/MS** | **Herb Source** |
| --- | --- | --- | --- | --- | --- | --- | --- | --- | --- |
| 8 | Valine^#^ | 118.086 | [M+H]^+^ | -2.2 | 2.54 | 14740 | C_5_H_11_NO_2_ | 118.0858[M +H]^+^ 72.0809[M+H-HCOOH]^+^ | IR |
| 9 | 5-Hydroxyoxindole | 150.0546 | [M+H]^+^ | -2.4 | 3.03 | 24455 | C_8_H_7_NO_2_ | 150.0548[M +H]^+^ 133.0521[M +H- OH]^+^ 132.0451[M +H-H_2_O]+ | IR |
| 10 | Indole-4-carboxaldehyde | 146.0599 | [M+H]^+^ | -0.9 | 3.94 | 12720 | C_9_H_7_NO | 146.0602[M+H]^+^ 118.0644[M+H-CO]^+^ 117.0569[M+H-CHO]^+^ | BR, IR, ASH |
| 11 | Chlorogenic acid* | 355.102 | [M+H]^+^ | -0.9 | 5.1 | 8203 | C_16_H_18_O_9_ | 355.1014[M+H]^+^ 337.0905[M+H-H_2_O]^+^  176.0679[M+H-C_9_H_7_O_4_]^+^ 163.0386[M+H-C_7_H_12_O_6_]^+^ 135.0436[M+H-C_7_H_12_O_6_-CO]^+^ | BR, ASH |
| 12 | Clemastanin B | 683.2508 | [M-H]^-^ | -7.1 | 6.39 | 6621 | C_32_H_44_O_16_ | 683.2521[M-H]^-^ 329.1444[M-H-C_13_H_22_O_11_]^-^ 521.2032[M-H-C_6_H_10_O5]^-^ 359.1540[M-H-C_12_H_20_O_10_]^-^ | IR |

Table S1 (Continued)

| **NO** | **Identification** | **Observed （m/z）** | **Adducts** | **Mass error (ppm)** | **t_R_ (min)** | **Response** | **Formula** | **MS/MS** | **Herb Source** |
| --- | --- | --- | --- | --- | --- | --- | --- | --- | --- |
| 13 | Kaempferol-3-O-rutinoside | 593.1494 | [M-H]^-^ | -3 | 6.46 | 35744 | C_27_H_30_O_15_ | 593.1508[M-H]^-^ 473.1107[M-H-C_4_H_8_O_4_]-  383.0809[M-H-C_7_H_12_O_7_]^-^ 353.0719[M-H-C_8_H_16_O_8_]-  285.1260[M-H-C_6_H_10_O_5_-C_6_H_10_O_4_]^-^ | ASH |
| 14 | Kaempferol-3-*O*-rhamnoside | 431.1075 | [M-H]^-^ | -0.6 | 6.87 | 15860 | C_21_H_20_O_10_ | 431.1075[M-H]^-^ 285.1206[M-H-C_6_H_10_O_4_]^-^ | ASH |
| 15 | Quercetin-3-*O*-robinobioside | 611.1601 | [M+H]^+^ | -0.9 | 7.06 | 7772 | C_27_H_30_O_16_ | 633.1432[M+Na]^+^ 611.1602[M+H]^+^ 303.0499[M+H-C_6_H_10_O_5_-C_6_H_10_O_4_]^+^ 465.1024[M+H-C_6_H_10_O_4_]^+^ 449.1068[M+H- C_6_H_10_O_5_]^+^ | ASH |
| 16 | Deoxyvasicinone | 187.0863 | [M+H]^+^ | -1.4 | 7.28 | 67971 | C_11_H_10_N_2O_ | 187.0862[M+H]^+^ 146.0597[M+H-CN-CH_3_]^+^ 116.0494[M+H-C_3_H_5_NO]^+^ 133.0524[M+H-C_3_H_4_N]^+^ | IR |

Table S1 (Continued)

| **NO** | **Identification** | **Observed （m/z）** | **Adducts** | **Mass error (ppm)** | **t_R_ (min)** | **Response** | **Formula** | **MS/MS** | **Herb Source** |
| --- | --- | --- | --- | --- | --- | --- | --- | --- | --- |
| 17 | Scopoletin * | 193.0493 | [M+H]^+^ | -1 | 7.91 | 11491 | C_10_H_8_O_4_ | 193.0493[M+H]^+^ 163.0388[M+H-CH_2_O]^+^ 151.0389[M+H-CH_2_-CO]^+^ 147.0439[M+H-H_2_O-CO]^+^  119.0480[M+H-H_2_O-CO-CO]^+^ | BR, ASH |
| 18 | Indigoticoside A | 521.2028 | [M-H]^-^ | -0.1 | 8.15 | 5908 | C_26_H_34_O_11_ | 521.2033[M-H]^+^ 329.1444[M-H-C_7_H_12_O_6_]^+^ 359.1540[M-H-C_6_H_10_O_5_]^+^ | IR |
| 19 | Rutin * | 611.1601 | [M+H]^+^ | -0.9 | 8.37 | 15200 | C_27_H_30_O_16_ | 611.1602[M+H] ^+^  633.1432[M+Na] ^+^ 465.1024[M+H-C_6_H_10_O_4_] ^+^  303.0499[M+H-C_6_H_10_O_5_-C_6_H_10_O_4_] ^+^ 285.0392[M+H-C_6_H_10_O_5_-C_6_H_10_O_4_-H_2_O] ^+^ 257.0450[M+H-C_6_H_10_O_5_-C_6_H_10_O_4_-H_2_O-CO] ^+^ 229.0501[M+H-C_6_H_10_O_5_-C_6_H_10_O_4_-H_2_O-2CO] ^+^  165.0170[M+H-C_6_H_10_O_5_-C_6_H_10_O_4_-C_7_H_6_O_3_] ^+^  153.0170[M+H-C_6_H_10_O_5_-C_6_H_10_O_4_-C_8_H_6_O_3_] ^+^  137.0241[M+H-C_6_H_10_O_5_-C_6_H_10_O_4_-C_8_H_6_O_4_] ^+^ | BR, MB, ASH |

Table S1 (Continued)

| **NO** | **Identification** | **Observed （m/z）** | **Adducts** | **Mass error (ppm)** | **t_R_ (min)** | **Response** | **Formula** | **MS/MS** | **Herb Source** |
| --- | --- | --- | --- | --- | --- | --- | --- | --- | --- |
| 20 | Hyperoside * | 465.1023 | [M+H]^+^ | -1 | 8.37 | 3837 | C_21_H_20_O_12_ | 465.1029[M+H] ^+^  303.0501[M+H-C_6_H_10_O_5_] ^+^  285.0381[M+H-C_6_H_10_O_5_-C_6_H_10_O_4_-H_2_O] ^+^ 257.0439[M+H-C_6_H_10_O_5_-C_6_H_10_O_4_-H_2_O-CO] ^+^  165.0188[M+H-C_6_H_10_O_5_-C_7_H_6_O_3_] ^+^  153.0188[M+H-C_6_H_10_O_5_-C_8_H_6_O_3_] ^+^  137.0241[M+H-C_6_H_10_O_5_-C_8_H_6_O_4_] ^+^ | BR，ASH |
| 21 | Vitexin * | 433.1135 | [M+H]^+^ | 3.0 | 8.38 | 588068 | C_21_H_20_O_10_ | 433.1135[M+H]^+^  415.1029[M+H-H_2_O]^+^  397.0942[M+H-2H_2_O]^+^  379.0851[M+H-3H_2_O]^+^  271.0614[M+H-H_2_O-C_6_H_9_O_4_]^+^ | MB |
| 22 | Isoquercitrin | 465.1013 | [M+H]^+^ | 3.8 | 8.59 | 26635 | C_21_H_20_O_12_ | 487.0852[M+Na] ^+^ 465.1029[M+H] ^+^  303.0499[M+H-C_6_H_10_O_5_] ^+^  165.0170[M+H-C_6_H_10_O_5_-C_7_H_6_O_3_] ^+^  153.0182[M+H-C_14_H_16_O_8_] ^+^  137.0241[M+H-C_14_H_16_O_9_] ^+^ | BR, ASH, MB |

Table S1 (Continued)

| **NO** | **Identification** | **Observed （m/z）** | **Adducts** | **Mass error (ppm)** | **t_R_ (min)** | **Response** | **Formula** | **MS/MS** | **Herb Source** |
| --- | --- | --- | --- | --- | --- | --- | --- | --- | --- |
| 23 | Isoquercitrin^#^ | 465.1013 | [M+H]^+^ | -3 | 8.66 | 1555 | C_21_H_20_O_12_ | 487.0852[M+Na] ^+^ 465.1029[M+H] ^+^  303.0499[M+H-C_6_H_10_O_5_] ^+^  165.0170[M+H-C_6_H_10_O_5_-C_7_H_6_O_3_] ^+^  153.0182[M+H-C_14_H_16_O_8_] ^+^  137.0241[M+H-C_14_H_16_O_9_] ^+^ | BR, ASH |
| 24 | (+)-isolariciresinol 9'-O-glucoside | 521.2028 | [M+H]^+^ | -0.1 | 8.77 | 21749 | C_26_H_34_O_11_ | 521.2033[M-H]^+^ 329.1444[M-H-C_7_H_12_O_6_]^+^ 359.1540[M-H-C_6_H_10_O_5_]^+^ | IR |
| 25 | 4,5-dicaffeoylquinic acid | 517.1328 | [M+H]^+^ | 3 | 9.47 | 6799 | C_25_H_24_O_12_ | 517.1328[M+H]^+^  499.1232[M+H-H_2_O]^+^ 355.1031[M+H-C_9_H_6_O_3_]^+^ 163.0389[M+H-C_9_H_9_O_3_-C_7_H_12_O_6_]^+^ 135.0437[M+H-C_9_H_9_O_3_-C_7_H_12_O_6_-CO]^+^ | ASH |

Table S1 (Continued)

| NO | Identification | Observed （m/z） | Adducts | Mass error (ppm) | tR (min) | Response | Formula | MS/MS | Herb Source |
| --- | --- | --- | --- | --- | --- | --- | --- | --- | --- |
| 26 | Quercetin^#^ | 303.0495 | [M+H]^+^ | -1.3 | 9.48 | 5078 | C_15_H_10_O_7_ | 303.0501[M+H] ^+^ 285.0392[M+H-H_2_O] ^+^ 257.0450[M+H-H_2_O-CO] ^+^ 229.0501[M+H-H_2_O-2CO] ^+^  165.0170[M+H-C_7_H_6_O_3_] ^+^  153.0170[M+H-C_8_H_6_O_3_] ^+^  137.0241[M+H-C_8_H_6_O_4_] ^+^ | BR, ASH |
| 27 | Narcissin | 625.1759 | [M+H]^+^ | -0.7 | 9.66 | 4144 | C_28_H_32_O_16_ | 625.1765[M+H] ^+^ 647.1588[M+Na] ^+^ 479.1172[M+H-C_6_H_10_O_4_] ^+^ 317.0654[M+H-C_6_H_10_O_5_-C_6_H_10_O_4_] ^+^ | BR, ASH |
| 28 | Capillarisin | 317.0648 | [M+H]^+^ | -2.4 | 9.66 | 5921 | C_16_H_12_O_7_ | 317.0653[M+H]^+^ 273.0387[M+H-C_6_H_4_O]^+^ 303.0495[M+H-CH_2_]^+^ 302.0415[M+H-CH_3_]^+^ 285.0388[M+H-CH_3_OH]^+^ 301.0320[M+H-CH_4_]^+^ | ASH |

Table S1 (Continued)

| NO | Identification | Observed （m/z） | Adducts | Mass error (ppm) | tR (min) | Response | Formula | MS/MS | Herb Source |
| --- | --- | --- | --- | --- | --- | --- | --- | --- | --- |
| 29 | Scoparone * | 207.0647 | [M+H]^+^ | -2.2 | 10.45 | 1491 | C_11_H_10_O_4_ | 207.0651[M+H]^+^  192.0418[M+H-CH_3_]^+^  179.0708[M+H-CO]^+^  164.0425[M+H-CH_3_-CO]^+^  136.0497[M+H-CH_3_-2CO]^+^  163.0375[M+H-CO_2_]^+^  151.0759[M+H-2CO]^+^ | BR, ASH |
| 30 | Quercetin * | 303.0497 | [M+H]^+^ | -0.7 | 12.21 | 6733 | C_15_H_10_O_7_ | 303.0501[M+H]^+^ 285.0392[M+H-H_2_O]^+^ 257.0450[M+H-H_2_O-CO]^+^ 229.0501[M+H-H_2_O-2CO]^+^  165.0170[M+H-C_7_H_6_O_3_]^+^  153.0170[M+H-C_8_H_6_O_3_]^+^  137.0241[M+H-C_8_H_6_O_4_]^+^ | BR |
| 31 | Guamarolin | 357.1326 | [M+H]^+^ | -1.9 | 13.86 | 31430 | C_20_H_20_O_6_ | 357.1326[M+H]^+^  233.0820[M+H-C_7_H_8_O_2_]^+^  147.0439[M+H-C_11_H_14_O_4_]^+^  137.0598[M+H-C_12_H_12_O_4_]^+^ | BR |

Table S1 (Continued)

| NO | Identification | Observed （m/z） | Adducts | Mass error (ppm) | tR (min) | Response | Formula | MS/MS | Herb Source |
| --- | --- | --- | --- | --- | --- | --- | --- | --- | --- |
| 32 | 5,7-Dimethoxycoumarin | 207.0648 | [M+H]^+^ | -1.9 | 15.37 | 5777 | C_11_H_10_O_4_ | 207.0651[M+H]^+^ 164.0466[M+H-CH_3_-CO]^+^  147.0446[M+H-C_3_H_4_O_2_]^+^  119.0480[M+H-C_3_H_4_O_3_]^+^ | BR |
| 33 | Saikosaponin F | 929.5465 | [M+H]^+^ | -0.4 | 16.11 | 144718 | C_48_H_80_O_17_ | 929.5465[M+H]^+^ 951.5293[M+Na]^+^ 911.5343[M+H-H_2_O]^+^ 893.5239[M+H-2H_2_O]^+^ 603.4248[M+H-C_6_H_10_O_5_-C_6_H_10_O_4_-H_2_O]^+^  585.4140[M+H-C_6_H_10_O_5_-C_6_H_10_O_4_-2H_2_O]^+^  423.3614[M+H-2C_6_H_10_O_5_-C_6_H_10_O_4_-2H_2_O] ^+^ | BR |
| 34 | Glycohyodeoxycholic acid | 450.3127 | [M-H]^-^ | -0.2 | 16.17 | 94800 | C_26_H_43_NO_5_ | 448.3127[M-H]^-^ 404.3215[M-H-CO_2_]^-^ 386.3143[M-H-H_2_O-CO_2_]^-^ | PFS |
| 35 | Henridilactone A | 543.222 | [M+H]^+^ | -1 | 16.53 | 7720 | C_29_H_34_O_10_ | 543.2214[M+H]^+^ 525.2095[M+H-H_2_O]^+^ 507.2065[M+H-2H_2_O]^+^  497.2170[M+H-CO_2_]^+^  440.2207[M+H-CO2-CH_3_CO_2_]^+^ | SCF |

Table S1 (Continued)

| **NO** | **Identification** | **Observed （m/z）** | **Adducts** | **Mass error (ppm)** | **t_R_ (min)** | **Response** | **Formula** | **MS/MS** | **Herb Source** |
| --- | --- | --- | --- | --- | --- | --- | --- | --- | --- |
| 36 | Schisandrol A^#^ | 433.2208 | [M+H]^+^ | -3 | 17.2 | 14925 | C_24_H_32_O_7_ | 433.2202[M+H]^+^ 415.2097[M+H-H_2_O]^+^ 400.1887[M+H-H_2_O-CH_3_]^+^  384.1940[M+H-H_2_O-OCH_3_]^+^ 346.1378[M+H-H_2_O-C_5_H_9_]^+^ 353.1750[M+H-H_2_O-2OCH_3_]^+^ 369.1712[M+H-H_2_O-OCH_3_-CH_3_]^+^ 338.1511[M+H-H_2_O-2OCH_3_-CH_3_]^+^ | SCF |
| 37 | Hyocholic acid | 407.2876 | [M-H]^-^ | -1.9 | 17.64 | 7120 | C_24_H_40_O_5_ | 407.2876[M-H]^-^ 389.2740[M-H-H_2_O]^-^ 371.2637[M-H-2H_2_O]^-^  353.2527[M-H-3H_2_O]^-^ | PFS |
| 38 | Saikosaponin A | 781.4723 | [M+H]^+^ | -1.2 | 18.57 | 23879 | C_42_H_68_O_13_ | 781.4738[M+H]^+^  803.4558[M+Na]^+^ 601.4100[M+H-C_6_H_10_O_5_-H_2_O]^+^ 583.3995[M+H-C_6_H_10_O_5_-2H_2_O]^+^  455.3526[M+H-C_6_H_10_O_5_-C_6_H_10_O_4_-H_2_O]^+^ 437.3420[M+H-C_6_H_10_O_5_-C_6_H_10_O_4_-2H_2_O]^+^ | BR |

Table S1 (Continued)

| **NO** | **Identification** | **Observed （m/z）** | **Adducts** | **Mass error (ppm)** | **t_R_ (min)** | **Response** | **Formula** | **MS/MS** | **Herb Source** |
| --- | --- | --- | --- | --- | --- | --- | --- | --- | --- |
| 39 | Schisandrol A | 433.2215 | [M+H]^+^ | -1.3 | 18.67 | 401624 | C_24_H_32_O_7_ | 433.2202[M+H]^+^  455.2016[M+Na]^+^ 415.2097[M+H-H_2_O]^+^ 346.1378[M+H-H_2_O-C_5_H_9_]^+^ 400.1887[M+H-H_2_O-CH_3_]^+^ 384.1940[M+H-H_2_O-OCH_3_]^+^ 369.1712[M+H-H_2_O-OCH_3_-CH_3_]^+^ 338.1511[M+H-H_2_O-OCH_3_-CH_3_]^+^ 323.1318[M+H-H2O-OCH_3_-2CH_3_]+ | SCF |
| 40 | Saikosaponin B2 * | 781.4738 | [M+H]^+^ | 0.7 | 18.70 | 364669 | C_42_H_68_O_13_ | 781.4738[M+H]^+^  803.4558[M+Na]^+^ 601.4100[M+H-C_6_H_10_O_5_-H_2_O]^+^ 583.3995[M+H-C_6_H_10_O_5_-2H_2_O]^+^  455.3526[M+H-C_6_H_10_O_5_-C_6_H_10_O_4_-H_2_O]^+^ 437.3420[M+H-C_6_H_10_O_5_-C_6_H_10_O_4_-2H_2_O]^+^ | BR |
| 41 | Glycochenodeoxycholic acid | 450.3120 | [M-H]^-^ | 0.1 | 19.28 | 166945 | C_26_H_43_NO_5_ | 448.3120[M-H]^-^ 404.3215[M-H-CO_2_]^-^ 386.3143[M-H-H_2_O-CO_2_]^-^ | PFS |

Table S1 (Continued)

| **NO** | **Identification** | **Observed （m/z）** | **Adducts** | **Mass error (ppm)** | **t_R_ (min)** | **Response** | **Formula** | **MS/MS** | **Herb Source** |
| --- | --- | --- | --- | --- | --- | --- | --- | --- | --- |
| 42 | Hyodeoxycholic acid * | 391.2852 | [M-H]^-^ | 1.0 | 19.35 | 266725 | C_24_H_40_O_4_ | 391.2852[M-H]^-^  783.5755[2M-H]^-^  373.2700[M-H-H_2_O]^-^  355.2675[M-H-2H_2_O]^-^ | PFS |
| 43 | Gomisin D | 548.2481 | [M+H]^+^ | -1.7 | 19.9 | 80713 | C_28_H_34_O_10_ | 531.2200[M+H]^+^ 485.2184[M+H-H_2_O]^+^ 401.1588[M+H-C_6_H_10_O_3_]^+^ 383.1507[M+H-C_6_H_10_O_3_-H_2_O]^+^ 341.1039[M+H-C_6_H_10_O_3_-H_2_O-C_3_H_6_]^+^ | SCF |
| 44 | Gomisin J | 389.1993 | [M+H]^+^ | -0.4 | 20.34 | 286126 | C_22_H_28_O_6_ | 389.1993[M+H]^+^ 319.1182[M+H-C_5_H_10_]^+^ 357.1739[M+H-CH_3_OH]+ 325.1440[M+H-2CH_3_OH]+ 287.0910[M+H-CH_3_OH-C_5_H_10_]^+^ 227.0690[M+H-2CH_3_OH-C_5_H_10_-CO]^+^ | SCF |

Table S1 (Continued)

| **NO** | **Identification** | **Observed （m/z）** | **Adducts** | **Mass error (ppm)** | **t_R_ (min)** | **Response** | **Formula** | **MS/MS** | **Herb Source** |
| --- | --- | --- | --- | --- | --- | --- | --- | --- | --- |
| 45 | Schisandrol B * | 416.1823 | [M+H]^+^ | -1.6 | 20.59 | 97473 | C_23_H_28_O_7_ | 417.1870[M+H]^+^ 369.1712[M+H-H_2_O-CH_2_O]^+^ 399.1849[M+H-H_2_O]^+^ 384.1598[M+H-H_2_O-CH_3_]^+^ 357.1326[M+H-H_2_O-C_3_H_6_]^+^ 353.1422[M+H-H_2_O-CH_3_-OCH_3_]^+^  343.1147[M+H-H_2_O-C_4_H_8_]^+^  341.1685[M+H-H_2_O-C_2_H_2_O_2_]^+^ 337.1402[M+H-H_2_O-CH_2_O-CH_3_OH]^+^ 330.1068[M+H-H_2_O-C_5_H_9_]^+^ 299.0943[M+H-H_2_O-C_5_H_9_-OCH_3_]^+^ | WWZ |
| 46 | 6''-O-Acetylsaikosaponin A | 823.4834 | [M+H]^+^ | -0.5 | 22.65 | 25242 | C_44_H_70_O_14_ | 823.4843[M+H]^+^ 845.4663[M+Na]^+^ 601.4091[M+H-C_6_H_10_O_5_-CH_3_COOH]^+^ 583.3973[M+H-C_6_H_10_O_5_-CH_3_COOH-H_2_O]^+^  455.3512[M+H-C_6_H_10_O_5_-C_6_H_10_O_4_-CH_3_COOH]^+^ 437.34028[M+H-C_6_H_10_O_5_-C_6_H_10_O_4_-CH_3_COOH-H_2_O]^+^ 419.3297[M+H-C_6_H_10_O_5_-C_6_H_10_O_4_-CH_3_COOH-2H_2_O]^+^ | BR |

Table S1 (Continued)

| **NO** | **Identification** | **Observed （m/z）** | **Adducts** | **Mass error (ppm)** | **t_R_ (min)** | **Response** | **Formula** | **MS/MS** | **Herb Source** |
| --- | --- | --- | --- | --- | --- | --- | --- | --- | --- |
| 47 | Pregomisin | 391.2111 | [M+H]^+^ | -1.2 | 23.59 | 100377 | C_22_H_30_O_6_ | 391.2088[M+H]^+^ 237.1455[M+H-C_8_H_10_O_3_]^+^ 205.1264[M+H-C_8_H_10_O_3_-CH_3_OH]^+^ 167.0704[M+H-C_13_H_20_O_3_]^+^ | SCF |
| 48 | Angeloylgomisin H | 501.2457 | [M+H]^+^ | -0.8 | 24.58 | 1285051 | C_28_H_36_O_8_ | 501.2457[M+H]^+^ 386.1734[M+H-C_4_H_7_COOH-CH_3_]^+^ 401.1934[M+H-C_4_H_7_COOH]^+^ 483.2393[M+H-H_2_O]^+^ 370.1785[M+H-C_4_H_7_COOH-OCH_3_]^+^ 355.1552[M+H-C_4_H_7_COOH-OCH_3_-CH_3_]^+^ 523.2303[M+Na]^+^ | SCF |
| 49 | Chenodeoxycholic acid* | 391.2852 | [M-H]^-^ | 1.2 | 25.28 | 5749 | C_24_H_40_O_4_ | 391.2852[M-H]^-^ 783.5755[2M-H]^-^ 373.2785[M-H-H_2_O]^-^ 355.2675[M-H-2H_2_O]^-^ | PFS |
| 50 | Benzoylgomisin H | 523.2315 | [M+H]^+^ | -2.2 | 25.46 | 34280 | C_30_H_34_O_8_ | 523.2303[M+H]^+^ 505.2221[M+H-H_2_O]^+^ 401.1937[M+H-C_6_H_5_COOH]^+^ 370.1785[M+H-C_6_H_5_COOH-OCH_3_]^+^ | SCF |

Table S1 (Continued)

| **NO** | **Identification** | **Observed （m/z）** | **Adducts** | **Mass error (ppm)** | **t_R_ (min)** | **Response** | **Formula** | **MS/MS** | **Herb Source** |
| --- | --- | --- | --- | --- | --- | --- | --- | --- | --- |
| 51 | Benzoylgomisin Q | 553.2407 | [M+H]^+^ | -4.6 | 25.94 | 36046 | C_31_H_36_O_9_ | 553.2291[M+H]^+^ 431.2052[M+H-C_6_H_5_COOH]^+^ 387.1779[M+H-C_6_H_5_COOH-C_2_H_4_O]^+^ 356.1597[M+H-C_6_H_5_COOH-C_2_H_4_O-OCH_3_]^+^ 341.1362[M+H-C_6_H_5_COOH -C_2_H_4_O-OCH_3_-CH3]^+^ | SCF |
| 52 | Gomisin G | 537.2141 | [M+H]^+^ | 0.1 | 26.49 | 399638 | C_31_H_36_O_9_ | 415.1741[M+H-C_6_H_5_COOH]+ 371.1451[M+H-C_6_H_5_COOH-C_2_H_4_O]^+^ 373.1244[M+H-C_6_H_5_COOH -C_3_H_6_]^+^ 397.1638[M+H-C_6_H_5_COOH -H_2_O]^+^ 340.1290[M+H-C_6_H_5_COOH -C_2_H_4_O-OCH_3_]^+^ 356.1267[M+H-C_6_H_5_COOH -C_2_H_4_O-CH_3_]^+^ 554.2460[M+NH_4_]^+^ 559.1909[M+Na]^+^ | SCF |

Table S1 (Continued)

| **NO** | **Identification** | **Observed （m/z）** | **Adducts** | **Mass error (ppm)** | **t_R_ (min)** | **Response** | **Formula** | **MS/MS** | **Herb Source** |
| --- | --- | --- | --- | --- | --- | --- | --- | --- | --- |
| 53 | Gomisin G^#^ | 537.2141 | [M+H]^+^ | -1.3 | 26.69 | 16129 | C_31_H_36_O_9_ | 537.2141[M+H]^+^  415.1741[M+H-C_6_H_5_COOH]^+^ 371.1451[M+H-C_6_H_5_COOH-C_2_H_4_O]^+^ 373.1244[M+H-C_6_H_5_COOH-C_3_H_6_]^+^ 385.1617[M+H-C_6_H_5_COOH-CH_2_O]^+^ 340.1290[M+H-C_6_H_5_COOH-C_2_H_4_O-OCH_3_]^+^ 325.1125[M+H-C_6_H_5_COOH-C_2_H_4_O-OCH_3_-CH_3_]^+^ 356.1267[M+H-C_6_H_5_COOH-C_2_H_4_O-CH_3_]^+^ | SCF |
| 54 | Gomisin K1 or Gomisin K2 | 403.2113 | [M+H]^+^ | -0.4 | 27.08 | 159308 | C_23_H_30_O_6_ | 403.2163[M+H]^+^ 333.1360[M+H-C_5_H_10_]^+^ 371.1872[M+H-CH_3_OH]^+^ 340.1692[M+H-CH_3_OH-OCH_3_]^+^ 302.1146[M+H-OCH_3_-C_5_H_10_]^+^ 301.1062[M+H-CH_3_OH-C_5_H_10_]^+^ 287.0910[M+H-OCH_3_-C_5_H_10_-CH_3_]^+^ 227.0690[M+H-OCH_3_-C_5_H_10_-CH_3_-C_2_H_4_O_2_]^+^ | SCF |

Table S1 (Continued)

| **NO** | **Identification** | **Observed （m/z）** | **Adducts** | **Mass error (ppm)** | **t_R_ (min)** | **Response** | **Formula** | **MS/MS** | **Herb Source** |
| --- | --- | --- | --- | --- | --- | --- | --- | --- | --- |
| 55 | Gomisin K1 or Gomisin K2 | 403.2111 | [M+H]^+^ | -0.9 | 27.29 | 703408 | C_23_H_30_O_6_ | 403.2163[M+H]^+^ 333.1360[M+H-C_5_H_10_]^+^ 371.1872[M+H-CH_3_OH]^+^ 340.1692[M+H-CH_3_OH-OCH_3_]^+^ 302.1146[M+H-OCH_3_-C5H10]+ 301.1062[M+H-CH_3_OH-C_5_H_10_]^+^ 287.0910[M+H-OCH_3_-C_5_H_10_-CH_3_]^+^ 227.0690[M+H-OCH_3_-C_5_H_10_-CH_3_-C_2_H_4_O_2_]^+^ | SCF |
| 56 | Schisantherin A^#^ | 537.2141 | [M+H]^+^ | 0.3 | 27.49 | 28093 | C_30_H_32_O_9_ | 537.2141[M+H]^+^ 559.1909[M+Na]^+^  415.1741[M+H-C_6_H_5_COOH]^+^ 371.1451[M+H-C_6_H_5_COOH-C_2_H_4_O]+ 373.1244[M+H-C_6_H_5_COOH-C_3_H_6_]+ 385.1617[M+H-C_6_H_5_COOH-CH_2_O]+ 397.1638[M+H-C_6_H_5_COOH-H_2_O]+ 340.1290[M+H-C_6_H_5_COOH-C_2_H_4_O-OCH_3_]^+^ 325.1125[M+H-C_6_H_5_COOH-C_2_H_4_O-OCH_3_-CH_3_]^+^ 356.1267[M+H-C_6_H_5_COOH-C_2_H_4_O-CH_3_]^+^ | SCF |

Table S1 (Continued)

| **NO** | **Identification** | **Observed （m/z）** | **Adducts** | **Mass error (ppm)** | **t_R_ (min)** | **Response** | **Formula** | **MS/MS** | **Herb Source** |
| --- | --- | --- | --- | --- | --- | --- | --- | --- | --- |
| 57 | Schisantherin A* | 537.2141 | [M+H]^+^ | 0.3 | 27.81 | 353970 | C_30_H_32_O_9_ | 537.2141[M+H]^+^ 559.1909[M+Na]^+^  415.1741[M+H-C_6_H_5_COOH]^+^ 371.1451[M+H-C_6_H_5_COOH-C_2_H_4_O]+ 373.1244[M+H-C_6_H_5_COOH-C_3_H_6_]+ 385.1617[M+H-C_6_H_5_COOH-CH_2_O]+ 397.1638[M+H-C_6_H_5_COOH-H_2_O]+ 340.1290[M+H-C_6_H_5_COOH-C_2_H_4_O-OCH_3_]^+^ 325.1125[M+H-C_6_H_5_COOH-C_2_H_4_O-OCH_3_-CH_3_]^+^ 356.1267[M+H-C_6_H_5_COOH-C_2_H_4_O-CH_3_]^+^ | SCF |
| 58 | Schisantherin A^#^ | 537.2141 | [M+H]^+^ | -3.9 | 27.99 | 90502 | C_30_H_32_O_9_ | 537.2141[M+H]^+^ 559.1909[M+Na]^+^  415.1741[M+H-C_6_H_5_COOH]^+^ 371.1451[M+H-C_6_H_5_COOH-C_2_H_4_O]+ 373.1244[M+H-C_6_H_5_COOH-C_3_H_6_]+ 385.1617[M+H-C_6_H_5_COOH-CH_2_O]+ 397.1638[M+H-C_6_H_5_COOH-H_2_O]+ 340.1290[M+H-C_6_H_5_COOH-C_2_H_4_O-OCH_3_]^+^ 325.1125[M+H-C_6_H_5_COOH-C_2_H_4_O-OCH_3_-CH_3_]^+^ 356.1267[M+H-C_6_H_5_COOH-C_2_H_4_O-CH_3_]^+^ | SCF |

Table S1 (Continued)

| **NO** | **Identification** | **Observed （m/z）** | **Adducts** | **Mass error (ppm)** | **t_R_ (min)** | **Response** | **Formula** | **MS/MS** | **Herb Source** |
| --- | --- | --- | --- | --- | --- | --- | --- | --- | --- |
| 59 | Schisanhenol * | 403.2112 | [M+H]^+^ | -0.8 | 28.47 | 337132 | C_23_H_30_O_6_ | 403.2163[M+H]^+^ 425.1974[M+Na]^+^  388.1922[M+H-CH_3_]^+^ 371.1872[M+H-CH_3_OH]^+^ 340.1612[M+H-CH_3_OH-OCH_3_]^+^ 302.1146[M+H-OCH_3_-C_5_H_10_]^+^ 301.1062[M+H-CH_3_OH-C_5_H_10_]^+^ | SCF |
| 60 | Gomisin M2 or Gomisin L1 /L2 | 387.1796 | [M+H]^+^ | -1.5 | 28.8 | 115389 | C_22_H_26_O_6_ | 387.1779[M+H]^+^ 409.1557[M+Na]^+^ 355.1552[M+H-CH_3_OH]^+^ 285.0750[M+H-CH_3_OH-C_5_H_10_]^+^ 227.0690[M+H-CH_3_OH-C_5_H_10_-C_2_H_2_O_2_]^+^ | SCF |
| 61 | Gomisin E | 515.2274 | [M+H]^+^ | -0.4 | 29.24 | 250246 | C_28_H_34_O_9_ | 515.2318[M+H]^+^ 385.1702[M+H-C_6_H_10_O_3_]^+^ 355.1552[M+H-C_6_H_10_O_3_-CH_2_O]^+^ 316.0915[M+H-C_6_H_10_O_3_-C_5_H_9_]_+_ 469.2245[M+H-CH_2_O_2_]_+_ | SCF |

Table S1 (Continued)

| **NO** | **Identification** | **Observed （m/z）** | **Adducts** | **Mass error (ppm)** | **t_R_ (min)** | **Response** | **Formula** | **MS/MS** | **Herb Source** |
| --- | --- | --- | --- | --- | --- | --- | --- | --- | --- |
| 62 | Linolenic acid | 279.2314 | [M+H]^+^ | -1.6 | 29.31 | 224876 | C_18_H_30_O_2_ | 279.2314[M+H]^+^ 209.1541[M+H-C_5_H_10_]^+^ 195.1399[M+H-C_6_H_12_]^+^  133.1009[M+H-C_10_H_18_O_2_]^+^  119.0852[M+H-C_9_H_20_O_2_]^+^ 105.0698[M+H-C_10_H_22_O_2_]^+^ 95.0855[M+H-C_11_H_20_O_2_]^+^ | BR, ASH |
| 63 | Gomisin M2 or Gomisin L1 /L2 | 387.1792 | [M+H]^+^ | -2.7 | 29.44 | 115894 | C_22_H_26_O_6_ | 387.1779[M+H]^+^ 409.1557[M+Na]^+^ 355.1552[M+H-CH_3_OH]^+^ 285.0750[M+H-CH_3_OH-C_5_H_10_]^+^ 227.0690[M+H-CH_3_OH-C_5_H_10_-C_2_H_2_O_2_]^+^ | SCF |
| 64 | Gomisin M2 or Gomisin L1 /L2 | 387.1796 | [M+H]^+^ | -1.6 | 29.7 | 192005 | C_22_H_26_O_6_ | 387.1779[M+H]^+^ 409.1557[M+Na]^+^ 355.1552[M+H-CH_3_OH]^+^ 285.0750[M+H-CH_3_OH-C_5_H_10_]^+^ 227.0690[M+H-CH_3_OH-C_5_H_10_-C_2_H_2_O_2_]^+^ | SCF |

Table S1 (Continued)

| **NO** | **Identification** | **Observed （m/z）** | **Adducts** | **Mass error (ppm)** | **t_R_ (min)** | **Response** | **Formula** | **MS/MS** | **Herb Source** |
| --- | --- | --- | --- | --- | --- | --- | --- | --- | --- |
| 65 | Schisandrin A * | 417.2268 | [M+H]^+^ | -1 | 30.54 | 3898313 | C_24_H_32_O_6_ | 439.2111[M+Na]^+^ 417.2316[M+H]^+^ 370.1785[M+H-C_2_H_7_O]^+^ 386.2077[M+H-OCH_3_]^+^ 316.1303[M+H-C_5_H_10_-OCH_3_]^+^ 301.1062[M+H-C_5_H_10_-OCH_3_-CH_3_]^+^ 285.1119[M+H-C_5_H_10_-2OCH_3_]^+^ 242.0925[M+H-C_5_H_10_-2OCH_3_-C_2_H_3_O]^+^ 227.0690[M+H-C_5_H_10_-2OCH_3_-C_2_H_3_O-CH_3_]^+^ 347.1539[M+H-C_5_H_10_]+ | SCF |
| 66 | Schisandrin B^#^ | 401.19 57 | [M+H]^+^ | -0.4 | 31.42 | 10188 | C_23_H_28_O_6_ | 401.2025[M+H]^+^  386.1734[M+H-CH_3_]^+^  371.1888[M+H-CH_2_O]^+^  331.1150[M+H-C_5_H_10_]^+^  300.0994[M+H-C_5_H_10_-OCH_3_]^+^  316.0993[M+H-C_5_H_1_0-CH_3_]^+^  423.1742[M+Na]^+^ | SCF |

Table S1 (Continued)

| **NO** | **Identification** | **Observed （m/z）** | **Adducts** | **Mass error (ppm)** | **t_R_ (min)** | **Response** | **Formula** | **MS/MS** | **Herb Source** |
| --- | --- | --- | --- | --- | --- | --- | --- | --- | --- |
| 67 | Schisandrin B * | 401.1954 | [M+H]^+^ | -1.2 | 31.66 | 5716694 | C_23_H_28_O_6_ | 401.2025[M+H]^+^ 423.1742[M+Na]^+^  386.1734[M+H-CH_3_]^+^ 371.1788[M+H-CH_2_O]^+^ 370.1785[M+H-OCH_3_]^+^ 331.1150[M+H-C_5_H_10_]^+^ 316.0993[M+H-C_5_H_10_-CH_3_]^+^ 300.0994[M+H-C_5_H_10_-OCH_3_]^+^ 285.0750[M+H-C_5_H_10_-OCH_3_-CH_3_]^+^ 273.1139[M+H-C_5_H_10_-C_2_H_2_O_2_]+ 227.0690[M+H-C_5_H_10_-OCH_3_-CH_3_-C_2_H_2_O_2_]^+^ | SCF |
| 68 | Schisandrin C * | 385.1642 | [M+H]^+^ | -0.8 | 32.24 | 593182 | C_22_H_24_O_6_ | 385.1617[M+H]^+^ 355.1552[M+H-CH_2_O]^+^ 315.0833[M+H-C_5_H_10_]^+^ 285.0750[M+H-C_5_H_10_-CH_2_O]^+^ 257.0789[M+H-C_5_H_10_-C_2_H_2_O_2_]^+^ 199.0750[M+H-C_5_H_10_-2C_2_H_2_O_2_]^+^ 184.0497[M+H-C_5_H_10_-2C_2_H_2_O_2_-CH_3_]^+^ | SCF |

Table S1 (Continued)

| **NO** | **Identification** | **Observed （m/z）** | **Adducts** | **Mass error (ppm)** | **t_R_ (min)** | **Response** | **Formula** | **MS/MS** | **Herb Source** |
| --- | --- | --- | --- | --- | --- | --- | --- | --- | --- |
| 69 | Kadsuric acid | 471.3466 | [M+H]^+^ | -0.5 | 35.1 | 47048 | C_30_H_46_O_4_ | 471.3453[M+H]^+^ 453.3405[M+H-H_2_O]^+^  427.3517[M+H-CO_2_]^+^ 145.1021[M+H-C_19_H_34_O_4_]^+^ | SCF |
| 70 | Schisanlactone D | 453.3361 | [M+H]^+^ | -0.4 | 35.14 | 19876 | C_30_H_44_O_3_ | 453.3405[M+H]^+^  435.3310[M+H-H_2_O]^+^  313.2724[M+H-C_8_H_12_O_2_]^+^  311.2371[M+H-C_8_H_14_O_2_]^+^  395.3647[M+H-C_2_H_2_O_2_]^+^  203.1801[M+H-C_15_H_21_O_3_]^+^  145.1021[M+H-C_19_H_31_O_3_]^+^ | SCF |
| 71 | Schisandronic acid | 455.3516 | [M+H]^+^ | -0.8 | 36.83 | 23900 | C_30_H_46_O_3_ | 455.3413[M+H]^+^  437.3465[M+H-H_2_O]^+^  145.1021[M+H-C_19_H_34_O_3_]^+^  133.1020[M+H-C_20_H_34_O_3_]^+^  159.1145[M+H-C_18_H_32_O_3_]^+^  173.1345[M+H-C_17_H_30_O_3_]^+^ | SCF |

a*: Confirmation in comparison with reference standards. Isomeric forms were distinguished by a^#^.

**Supplementary Table 2.** The 271 potential protein targets of Hugan Tablets in treating DILI.

| **No.** | **Gene Symbol** | **Uniprot ID** | **Description** |
| --- | --- | --- | --- |
| 1 | ABCB1 | P08183 | ATP-dependent translocase ABCB1 |
| 2 | ABCC1 | P33527 | Multidrug resistance-associated protein 1 |
| 3 | ABCG2 | Q9UNQ0 | Broad substrate specificity ATP-binding cassette transporter ABCG2 |
| 4 | ABL1 | P00519 | Tyrosine-protein kinase ABL1 |
| 5 | ACE | P12821 | Angiotensin-converting enzyme |
| 6 | ACHE | P22303 | Acetylcholinesterase |
| 7 | ACP1 | P24666 | Low molecular weight phosphotyrosine protein phosphatase |
| 8 | ADA | P00813 | Adenosine deaminase |
| 9 | ADH1B | P00325 | All-trans-retinol dehydrogenase [NAD(+)] ADH1B |
| 10 | ADH1C | P00326 | Alcohol dehydrogenase 1C |
| 11 | ADORA2A | P29274 | Adenosine receptor A2a |
| 12 | ADRB2 | P07550 | Beta-2 adrenergic receptor |
| 13 | AGTR1 | P30556 | Type-1 angiotensin II receptor |
| 14 | AHCY | P23526 | Adenosylhomocysteinase |
| 15 | AHR | P35869 | Aryl hydrocarbon receptor |
| 16 | AKR1A1 | P14550 | Aldo-keto reductase family 1 member A1 |
| 17 | AKT1 | P31749 | RAC-alpha serine/threonine-protein kinase |
| 18 | AKT2 | P31751 | RAC-beta serine/threonine-protein kinase |
| 19 | ALK | Q9UM73 | ALK tyrosine kinase receptor |
| 20 | ALOX5 | P09917 | Arachidonate 5-lipoxygenase |
| 21 | ALPL | P05186 | Alkaline phosphatase, tissue-nonspecific isozyme |
| 22 | ANPEP | P15144 | Aminopeptidase N |
| 23 | AOC3 | Q16853 | Membrane primary amine oxidase |

**Table S2 (Continued)**

|  | **No.** | | **Gene Symbol** | | **Uniprot ID** | | **Description** | |
| --- | --- | --- | --- | --- | --- | --- | --- | --- |
| 24 | | APEX1 | | P27695 | | DNA-(apurinic or apyrimidinic site) endonuclease | |  |
| 25 | | APP | | P05067 | | Amyloid-beta precursor protein | |  |
| 26 | | AR | | P10275 | | Androgen receptor | |  |
| 27 | | ARG1 | | P05089 | | Arginase-1 | |  |
| 28 | | ATM | | Q13315 | | Serine-protein kinase ATM | |  |
| 29 | | ATP12A | | P54707 | | Potassium-transporting ATPase alpha chain 2 | |  |
| 30 | | AURKA | | O14965 | | Aurora kinase A | |  |
| 31 | | AURKB | | Q96GD4 | | Aurora kinase B | |  |
| 32 | | AVPR2 | | P30518 | | Vasopressin V2 receptor | |  |
| 33 | | BCHE | | P06276 | | Cholinesterase | |  |
| 34 | | BCL2L1 | | Q07817 | | Bcl-2-like protein 1 | |  |
| 35 | | BLK | | P51451 | | Tyrosine-protein kinase Blk | |  |
| 36 | | BRAF | | P15056 | | Serine/threonine-protein kinase B-raf | |  |
| 37 | | BTK | | Q06187 | | Tyrosine-protein kinase BTK | |  |
| 38 | | C5AR1 | | P21730 | | C5a anaphylatoxin chemotactic receptor 1 | |  |
| 39 | | CASP1 | | P29466 | | Caspase-1 | |  |
| 40 | | CASP3 | | P42574 | | Caspase-3 | |  |
| 41 | | CASP7 | | P55210 | | Caspase-7 | |  |
| 42 | | CASP8 | | Q14790 | | Caspase-8 | |  |
| 43 | | CAT | | P04040 | | Catalase | |  |
| 44 | | CCNA2 | | P20248 | | Cyclin-A2 | |  |
| 45 | | CCNB1 | | P14635 | | G2/mitotic-specific cyclin-B1 | |  |
| 46 | | CCND1 | | P24385 | | G1/S-specific cyclin-D1 | |  |
| 47 | | CCNE1 | | P24864 | | G1/S-specific cyclin-E1 | |  |

**Table S2 (Continued)**

| **No.** | **Gene Symbol** | **Uniprot ID** | **Description** |
| --- | --- | --- | --- |
| 48 | CCR3 | P51677 | C-C chemokine receptor type 3 |
| 49 | CCR5 | P51681 | C-C chemokine receptor type 5 |
| 50 | CD22 | P20273 | B-cell receptor CD22 |
| 51 | CD38 | P28907 | ADP-ribosyl cyclase/cyclic ADP-ribose hydrolase 1 |
| 52 | CDK1 | P06493 | Cyclin-dependent kinase 1 |
| 53 | CDK2 | P24941 | Cyclin-dependent kinase 2 |
| 54 | CDK4 | P11802 | Cyclin-dependent kinase 4 |
| 55 | CDK5 | Q00535 | Cyclin-dependent-like kinase 5 |
| 56 | CDK6 | Q00534 | Cyclin-dependent kinase 6 |
| 57 | CES1 | P23141 | Liver carboxylesterase 1 |
| 58 | CETP | P11597 | Cholesteryl ester transfer protein |
| 59 | CFTR | P13569 | Cystic fibrosis transmembrane conductance regulator |
| 60 | CHKB | Q9Y259 | Choline/ethanolamine kinase |
| 61 | CHUK | O15111 | Inhibitor of nuclear factor kappa-B kinase subunit alpha |
| 62 | CNR1 | P21554 | Cannabinoid receptor 1 |
| 63 | COMT | P21964 | Catechol O-methyltransferase |
| 64 | CPT1A | P50416 | Carnitine O-palmitoyltransferase 1, liver isoform |
| 65 | CPT2 | P23786 | Carnitine O-palmitoyltransferase 2, mitochondrial |
| 66 | CSK | P41240 | Tyrosine-protein kinase CSK |
| 67 | CTSB | P07858 | Cathepsin B |
| 68 | CTSD | P07339 | Cathepsin D |
| 69 | CTSG | P08311 | Cathepsin G |
| 70 | CTSL | P07711 | Cathepsin L1 |
| 71 | CX3CR1 | P49238 | CX3C chemokine receptor 1 |

**Table S2 (Continued)**

| **No.** | **Gene Symbol** | **Uniprot ID** | **Description** |
| --- | --- | --- | --- |
| 72 | CXCR1 | P25024 | C-X-C chemokine receptor type 1 |
| 73 | CXCR2 | P25025 | C-X-C chemokine receptor type 2 |
| 74 | CYP17A1 | P05093 | Steroid 17-alpha-hydroxylase/17,20 lyase |
| 75 | CYP19A1 | P11511 | Aromatase |
| 76 | CYP1A2 | P05177 | Cytochrome P450 1A2 |
| 77 | CYP1B1 | Q16678 | Cytochrome P450 1B1 |
| 78 | CYP2A6 | P11509 | Cytochrome P450 2A6 |
| 79 | CYP2C19 | P33261 | Cytochrome P450 2C19 |
| 80 | CYP2C9 | P11712 | Cytochrome P450 2C9 |
| 81 | CYP3A4 | P08684 | Cytochrome P450 3A4 |
| 82 | DHFR | P00374 | Dihydrofolate reductase |
| 83 | DNMT1 | P26358 | DNA (cytosine-5)-methyltransferase 1 |
| 84 | DPP4 | P27487 | Dipeptidyl peptidase 4 |
| 85 | DRD2 | P14416 | D(2) dopamine receptor |
| 86 | DRD3 | P35462 | D(3) dopamine receptor |
| 87 | DRD4 | P21917 | D(4) dopamine receptor |
| 88 | EGFR | P00533 | Epidermal growth factor receptor |
| 89 | EIF2AK2 | P19525 | Interferon-induced, double-stranded RNA-activated protein kinase |
| 90 | ELANE | P08246 | Neutrophil elastase |
| 91 | EPHX1 | P07099 | Epoxide hydrolase 1 |
| 92 | EPHX2 | P34913 | Bifunctional epoxide hydrolase 2 |
| 93 | ERBB2 | P04626 | Receptor tyrosine-protein kinase erbB-2 |
| 94 | ERN1 | O75460 | Serine/threonine-protein kinase/endoribonuclease IRE1 |
| 95 | ESR1 | P03372 | Estrogen receptor |

**Table S2 (Continued)**

| **No.** | **Gene Symbol** | **Uniprot ID** | **Description** |
| --- | --- | --- | --- |
| 96 | ESR2 | Q92731 | Estrogen receptor beta |
| 97 | EZH2 | Q15910 | Histone-lysine N-methyltransferase EZH2 |
| 98 | F10 | P00742 | Coagulation factor X |
| 99 | F2 | P00734 | Prothrombin |
| 100 | F3 | P13726 | Tissue factor |
| 101 | F7 | P08709 | Coagulation factor VII |
| 102 | F9 | P00740 | Coagulation factor IX |
| 103 | FABP1 | P07148 | Fatty acid-binding protein, liver |
| 104 | FASN | P49327 | Fatty acid synthase |
| 105 | FGFR1 | P11362 | Fibroblast growth factor receptor 1 |
| 106 | FHIT | P49789 | Bis(5'-adenosyl)-triphosphatase |
| 107 | FLT1 | P17948 | Vascular endothelial growth factor receptor 1 |
| 108 | FLT3 | P36888 | Receptor-type tyrosine-protein kinase FLT3 |
| 109 | G6PD | P11413 | Glucose-6-phosphate 1-dehydrogenase |
| 110 | GABRG2 | P18507 | Gamma-aminobutyric acid receptor subunit gamma-2 |
| 111 | GAPDH | P04406 | Glyceraldehyde-3-phosphate dehydrogenase |
| 112 | GCK | P35557 | Hexokinase-4 |
| 113 | GLUL | P15104 | Glutamine synthetase |
| 114 | GSK3B | P49841 | Glycogen synthase kinase-3 beta |
| 115 | GSR | P00390 | Glutathione reductase, mitochondrial |
| 116 | GSTA1 | P08263 | Glutathione S-transferase A1 |
| 117 | HDAC1 | Q13547 | Histone deacetylase 1 |
| 118 | HIF1A | Q16665 | Hypoxia-inducible factor 1-alpha |
| 119 | HLA-A | P04439 | HLA class I histocompatibility antigen, A alpha chain |

**Table S2 (Continued)**

|  | **No.** | | **Gene Symbol** | | **Uniprot ID** | | **Description** | |
| --- | --- | --- | --- | --- | --- | --- | --- | --- |
| 120 | | HMGCR | | P04035 | | 3-hydroxy-3-methylglutaryl-coenzyme A reductase | |  |
| 121 | | HNF4A | | P41235 | | Hepatocyte nuclear factor 4-alpha | |  |
| 122 | | HRAS | | P01112 | | GTPase HRas | |  |
| 123 | | HRH1 | | P35367 | | Histamine H1 receptor | |  |
| 124 | | HSP90AA1 | | P07900 | | Heat shock protein HSP 90-alpha | |  |
| 125 | | HSP90B1 | | P14625 | | Endoplasmin | |  |
| 126 | | HSPA1A | | P0DMV8 | | Heat shock 70 kDa protein 1A | |  |
| 127 | | HSPA5 | | P11021 | | Endoplasmic reticulum chaperone BiP | |  |
| 128 | | HSPA8 | | P11142 | | Heat shock cognate 71 kDa protein | |  |
| 129 | | HTR1A | | P08908 | | 5-hydroxytryptamine receptor 1A | |  |
| 130 | | HTR2A | | P28223 | | 5-hydroxytryptamine receptor 2A | |  |
| 131 | | HTR2C | | P28335 | | 5-hydroxytryptamine receptor 2C | |  |
| 132 | | ICAM1 | | P05362 | | Intercellular adhesion molecule 1 | |  |
| 133 | | IGF1R | | P08069 | | Insulin-like growth factor 1 receptor | |  |
| 134 | | IGFBP3 | | P17936 | | Insulin-like growth factor-binding protein 3 | |  |
| 135 | | IL1B | | P01584 | | Interleukin-1 beta | |  |
| 136 | | IL2 | | P60568 | | Interleukin-2 | |  |
| 137 | | IL6 | | P05231 | | Interleukin-6 | |  |
| 138 | | INSR | | P06213 | | Insulin receptor | |  |
| 139 | | ITGA2B | | P08514 | | Integrin alpha-IIb | |  |
| 140 | | ITGA4 | | P13612 | | Integrin alpha-4 | |  |
| 141 | | ITGAL | | P20701 | | Integrin alpha-L | |  |
| 142 | | ITGB1 | | P05556 | | Integrin beta-1 | |  |
| 143 | | ITGB2 | | P05107 | | Integrin beta-2 | |  |

**Table S2 (Continued)**

|  | **No.** | | **Gene Symbol** | | **Uniprot ID** | | **Description** | |
| --- | --- | --- | --- | --- | --- | --- | --- | --- |
| 144 | | ITGB3 | | P05106 | | Integrin beta-3 | |  |
| 145 | | JAK1 | | P23458 | | Tyrosine-protein kinase JAK1 | |  |
| 146 | | JAK2 | | O60674 | | Tyrosine-protein kinase JAK2 | |  |
| 147 | | JUN | | P05412 | | Transcription factor AP-1 | |  |
| 148 | | KCNH2 | | Q12809 | | Potassium voltage-gated channel subfamily H member 2 | |  |
| 149 | | KDR | | P35968 | | Vascular endothelial growth factor receptor 2 | |  |
| 150 | | KIT | | P10721 | | Mast/stem cell growth factor receptor Kit | |  |
| 151 | | KMT2A | | Q03164 | | Histone-lysine N-methyltransferase 2A | |  |
| 152 | | LCK | | P06239 | | Tyrosine-protein kinase Lck | |  |
| 153 | | LDLR | | P01130 | | Low-density lipoprotein receptor | |  |
| 154 | | LGALS3 | | P17931 | | Galectin-3 | |  |
| 155 | | LYN | | P07948 | | Tyrosine-protein kinase Lyn | |  |
| 156 | | MAOA | | P21397 | | Amine oxidase [flavin-containing] A | |  |
| 157 | | MAOB | | P27338 | | Amine oxidase [flavin-containing] B | |  |
| 158 | | MAP2K1 | | Q02750 | | Dual specificity mitogen-activated protein kinase kinase 1 | |  |
| 159 | | MAPK1 | | P28482 | | Mitogen-activated protein kinase 1 | |  |
| 160 | | MAPK10 | | P53779 | | Mitogen-activated protein kinase 10 | |  |
| 161 | | MAPK14 | | Q16539 | | Mitogen-activated protein kinase 14 | |  |
| 162 | | MAPK3 | | P27361 | | Mitogen-activated protein kinase 3 | |  |
| 163 | | MAPK8 | | P45983 | | Mitogen-activated protein kinase 8 | |  |
| 164 | | MAPK9 | | P45984 | | Mitogen-activated protein kinase 9 | |  |
| 165 | | MAPT | | P10636 | | Microtubule-associated protein tau | |  |
| 166 | | MARS1 | | P56192 | | Methionine--tRNA ligase, cytoplasmic | |  |
| 167 | | MB | | P02144 | | Myoglobin | |  |

**Table S2 (Continued)**

| **No.** | | **Gene Symbol** | | **Uniprot ID** | | **Description** | |
| --- | --- | --- | --- | --- | --- | --- | --- |
| 168 | MCL1 | | Q07820 | | Induced myeloid leukemia cell differentiation protein Mcl-1 | |  |
| 169 | MDM2 | | Q00987 | | E3 ubiquitin-protein ligase Mdm2 | |  |
| 170 | MET | | P08581 | | Hepatocyte growth factor receptor | |  |
| 171 | MIF | | P14174 | | Macrophage migration inhibitory factor | |  |
| 172 | MMP1 | | P03956 | | Interstitial collagenase | |  |
| 173 | MMP14 | | P50281 | | Matrix metalloproteinase-14 | |  |
| 174 | MMP2 | | P08253 | | 72 kDa type IV collagenase | |  |
| 175 | MMP3 | | P08254 | | Stromelysin-1 | |  |
| 176 | MMP7 | | P09237 | | Matrilysin | |  |
| 177 | MMP9 | | P14780 | | Matrix metalloproteinase-9 | |  |
| 178 | MPO | | P05164 | | Myeloperoxidase | |  |
| 179 | MTOR | | P42345 | | Serine/threonine-protein kinase mTOR | |  |
| 180 | MTTP | | P55157 | | Microsomal triglyceride transfer protein large subunit | |  |
| 181 | NAMPT | | P43490 | | Nicotinamide phosphoribosyltransferase | |  |
| 182 | NAT1 | | P18440 | | Arylamine N-acetyltransferase 1 | |  |
| 183 | NGFR | | P08138 | | Tumor necrosis factor receptor superfamily member 16 | |  |
| 184 | NOS1 | | P29475 | | Nitric oxide synthase, brain | |  |
| 185 | NOS2 | | P35228 | | Nitric oxide synthase, inducible | |  |
| 186 | NOS3 | | P29474 | | Nitric oxide synthase, endothelial | |  |
| 187 | NOX4 | | Q9NPH5 | | NADPH oxidase 4 | |  |
| 188 | NQO1 | | P15559 | | NAD(P)H dehydrogenase [quinone] 1 | |  |
| 189 | NR1H4 | | Q96RI1 | | Bile acid receptor | |  |
| 190 | NR1I2 | | O75469 | | Nuclear receptor subfamily 1 group I member 2 | |  |
| 191 | NR1I3 | | Q14994 | | Nuclear receptor subfamily 1 group I member 3 | |  |

**Table S2 (Continued)**

|  | **No.** | | **Gene Symbol** | | **Uniprot ID** | | **Description** | |
| --- | --- | --- | --- | --- | --- | --- | --- | --- |
| 192 | | NR3C1 | | P04150 | | Glucocorticoid receptor | |  |
| 193 | | PARP1 | | P09874 | | Poly [ADP-ribose] polymerase 1 | |  |
| 194 | | PDE5A | | O76074 | | cGMP-specific 3',5'-cyclic phosphodiesterase | |  |
| 195 | | PDGFRA | | P16234 | | Platelet-derived growth factor receptor alpha | |  |
| 196 | | PDGFRB | | P09619 | | Platelet-derived growth factor receptor beta | |  |
| 197 | | PIK3CA | | P42336 | | Phosphatidylinositol 4,5-bisphosphate 3-kinase catalytic subunit alpha isoform | |  |
| 198 | | PIK3CG | | P48736 | | Phosphatidylinositol 4,5-bisphosphate 3-kinase catalytic subunit gamma isoform | |  |
| 199 | | PIK3R1 | | P27986 | | Phosphatidylinositol 3-kinase regulatory subunit alpha | |  |
| 200 | | PLAU | | P00749 | | Urokinase-type plasminogen activator | |  |
| 201 | | PPARA | | Q07869 | | Peroxisome proliferator-activated receptor alpha | |  |
| 202 | | PPARG | | P37231 | | Peroxisome proliferator-activated receptor gamma | |  |
| 203 | | PPARD | | Q03181 | | Peroxisome proliferator-activated receptor delta | |  |
| 204 | | PRKAB1 | | Q9Y478 | | 5'-AMP-activated protein kinase subunit beta-1 | |  |
| 205 | | PRKCA | | P17252 | | Protein kinase C alpha type | |  |
| 206 | | PRKCD | | Q05655 | | Protein kinase C delta type | |  |
| 207 | | PRKCZ | | Q05513 | | Protein kinase C zeta type | |  |
| 208 | | PTGS1 | | P23219 | | Prostaglandin G/H synthase 1 | |  |
| 209 | | PTGS2 | | P35354 | | Prostaglandin G/H synthase 2 | |  |
| 210 | | PTK2 | | Q05397 | | Focal adhesion kinase 1 | |  |
| 211 | | PTK2B | | Q14289 | | Protein-tyrosine kinase 2-beta | |  |
| 212 | | PTPN22 | | Q9Y2R2 | | Tyrosine-protein phosphatase non-receptor type 22 | |  |
| 213 | | PTPRC | | P08575 | | Receptor-type tyrosine-protein phosphatase C | |  |
| 214 | | PYGL | | P06737 | | Glycogen phosphorylase, liver form | |  |
| 215 | | QDPR | | P09417 | | Dihydropteridine reductase | |  |

**Table S2 (Continued)**

|  | **No.** | | **Gene Symbol** | | **Uniprot ID** | | **Description** | |
| --- | --- | --- | --- | --- | --- | --- | --- | --- |
| 216 | | RAF1 | | P04049 | | RAF proto-oncogene serine/threonine-protein kinase | |  |
| 217 | | RARA | | P10276 | | Retinoic acid receptor alpha | |  |
| 218 | | RARB | | P10826 | | Retinoic acid receptor beta | |  |
| 219 | | RARS1 | | P54136 | | Arginine--tRNA ligase, cytoplasmic | |  |
| 220 | | RBP4 | | P02753 | | Retinol-binding protein 4 | |  |
| 221 | | RELA | | Q04206 | | Transcription factor p65 | |  |
| 222 | | REN | | P00797 | | Renin | |  |
| 223 | | RET | | P07949 | | Proto-oncogene tyrosine-protein kinase receptor Ret | |  |
| 224 | | RPS6KA3 | | P51812 | | Ribosomal protein S6 kinase alpha-3 | |  |
| 225 | | SCARB1 | | Q8WTV0 | | Scavenger receptor class B member 1 | |  |
| 226 | | SCD | | O00767 | | Acyl-CoA desaturase | |  |
| 227 | | SCN1A | | P35498 | | Sodium channel protein type 1 subunit alpha | |  |
| 228 | | SCN5A | | Q14524 | | Sodium channel protein type 5 subunit alpha | |  |
| 229 | | SELE | | P16581 | | E-selectin | |  |
| 230 | | SELL | | P14151 | | L-selectin | |  |
| 231 | | SELP | | P16109 | | P-selectin | |  |
| 232 | | SERPINA6 | | P08185 | | Corticosteroid-binding globulin | |  |
| 233 | | SERPINE1 | | P05121 | | Plasminogen activator inhibitor 1 | |  |
| 234 | | SHBG | | P04278 | | Sex hormone-binding globulin | |  |
| 235 | | SHH | | Q15465 | | Sonic hedgehog protein | |  |
| 236 | | SLC10A1 | | Q14973 | | Sodium/bile acid cotransporter | |  |
| 237 | | SLC10A2 | | Q12908 | | Ileal sodium/bile acid cotransporter | |  |
| 238 | | SLC22A12 | | Q96S37 | | Solute carrier family 22 member 12 | |  |
| 239 | | SLC6A3 | | Q01959 | | Sodium-dependent dopamine transporter | |  |

**Table S2 (Continued)**

| **No.** | **Gene Symbol** | **Uniprot ID** | **Description** |
| --- | --- | --- | --- |
| 240 | SLC6A4 | P31645 | Sodium-dependent serotonin transporter |
| 241 | SMARCA4 | P51532 | Transcription activator BRG1 |
| 242 | SRC | P12931 | Proto-oncogene tyrosine-protein kinase Src |
| 243 | STAT3 | P40763 | Signal transducer and activator of transcription 3 |
| 244 | STAT6 | P42226 | Signal transducer and activator of transcription 6 |
| 245 | SYK | P43405 | Tyrosine-protein kinase SYK |
| 246 | TEK | Q02763 | Angiopoietin-1 receptor |
| 247 | TERT | O14746 | Telomerase reverse transcriptase |
| 248 | TGFB1 | P01137 | Transforming growth factor beta-1 proprotein |
| 249 | TGFBR2 | P37173 | TGF-beta receptor type-2 |
| 250 | TGM2 | P21980 | Protein-glutamine gamma-glutamyltransferase 2 |
| 251 | TLR4 | O00206 | Toll-like receptor 4 |
| 252 | TLR7 | Q9NYK1 | Toll-like receptor 7 |
| 253 | TLR8 | Q9NR97 | Toll-like receptor 8 |
| 254 | TLR9 | Q9NR96 | Toll-like receptor 9 |
| 255 | TNF | P01375 | Tumor necrosis factor |
| 256 | TNFRSF1A | P19438 | Tumor necrosis factor receptor superfamily member 1A |
| 257 | TOP1 | P11387 | DNA topoisomerase 1 |
| 258 | TOP2A | P11388 | DNA topoisomerase 2-alpha |
| 259 | TPMT | P51580 | Thiopurine S-methyltransferase |
| 260 | TPO | P07202 | Thyroid peroxidase |
| 261 | TTR | P02766 | Transthyretin |
| 262 | TYK2 | P29597 | Non-receptor tyrosine-protein kinase TYK2 |
| 263 | TYMP | P19971 | Thymidine phosphorylase |

**Table S2 (Continued)**

| **No.** | **Gene Symbol** | **Uniprot ID** | **Description** |
| --- | --- | --- | --- |
| 264 | TYMS | P04818 | Thymidylate synthase |
| 265 | TYR | P14679 | Tyrosinase |
| 266 | UGT2B7 | P16662 | UDP-glucuronosyltransferase 2B7 |
| 267 | VCAM1 | P19320 | Vascular cell adhesion protein 1 |
| 268 | VDR | P11473 | Vitamin D3 receptor |
| 269 | VEGFA | P15692 | Vascular endothelial growth factor A |
| 270 | XDH | P47989 | Xanthine dehydrogenase/oxidase |
| 271 | XIAP | P98170 | E3 ubiquitin-protein ligase XIAP |

**Supplementary Table 3. The 30 potentially active ingredients in Hugan Tablets.**

| **NO** | **CAS** | **Compounds** | **Molecular Formula** | **Herb Source** |
| --- | --- | --- | --- | --- |
| 1 | 62687-63-2 | saikosaponin F | C_48_H_80_O_17_ | BR |
| 2 | 112747-98-5 | clemastanin B | C_32_H_44_O_16_ | IR |
| 3 | 17650-84-9 | kaempferol-3-*O*-rutinoside | C_27_H_30_O_15_ | ASH |
| 4 | 52525-35-6 | quercetin-3-*O*-robinobioside | C_27_H_30_O_16_ | ASH |
| 5 | 153-18-4 | rutin | C_27_H_30_O_16_ | BR, MB, ASH |
| 6 | 143663-00-7 | indigoticoside A | C_26_H_34_O_11_ | IR |
| 7 | 20736-09-8 | saikosaponin A | C_42_H_68_O_13_ | BR |
| 8 | 57378-72-0 | 4,5-dicaffeoylquinic acid | C_25_H_24_O_12_ | ASH |
| 9 | 604-80-8 | narcissin | C_28_H_32_O_16_ | BR, ASH |
| 10 | 63358-12-3 | (+)-isolariciresinol 9'-O-glucoside | C_26_H_34_O_11_ | IR |
| 11 | 64340-46-1 | 6''-O-acetylsaikosaponin A | C_44_H_70_O_14_ | BR |
| 12 | 145374-38-5 | guamarolin | C_20_H_20_O_6_ | BR |
| 13 | 482-36-0 | hyperoside | C_21_H_20_O_12_ | BR，ASH |
| 14 | 482-39-3 | kaempferol-3-*O*-rhamnoside | C_21_H_20_O_10_ | ASH |
| 15 | 21637-25-2 | isoquercitrin | C_21_H_20_O_12_ | BR, ASH, MB |
| 16 | 66280-26-0 | pregomisin | C_22_H_30_O_6_ | SCF |
| 17 | 55511-14-3 | schisandronic acid | C_30_H_46_O_3_ | SCF |
| 18 | 120-08-1 | scoparone | C_11_H_10_O_4_ | BR, ASH |
| 19 | 62393-88-8 | kadsuric acid | C_30_H_46_O_4_ | SCF |
| 20 | 3681-93-4 | vitexin | C_21_H_20_O_10_ | MB |
| 21 | 58316-41-9 | saikosaponin B2 | C_42_H_68_O_13_ | BR |

**Table S3(Continued)**

| **NO** | **CAS** | **Compounds** | **Molecular Formula** | **Herb Source** |
| --- | --- | --- | --- | --- |
| 22 | 640-79-9 | glycochenodeoxycholic acid | C_26_H_43_NO_5_ | PFS |
| 23 | 547-75-1 | hyocholic acid | C_24_H_40_O_5_ | PFS |
| 24 | 463-40-1 | linolenic acid | C_18_H_30_O_2_ | BR, ASH |
| 25 | 474-25-9 | chenodeoxycholic acid | C_24_H_40_O_4_ | PFS |
| 26 | 92051-26-8 | schisanlactone D | C_30_H_44_O_3_ | SCF |
| 27 | 83-49-8 | hyodeoxycholic acid | C_24_H_40_O_4_ | PFS |
| 28 | 327-97-9 | chlorogenic acid | C_16_H_18_O_9_ | BR, ASH |
| 29 | 56365-38-9 | capillarisin | C_16_H_12_O_7_ | ASH |
| 30 | 117-39-5 | quercetin | C_15_H_10_O_7_ | BR |

**Supplementary Table 4.** The detailed information of the docking scores above the cut-off values.

|  | **GAPDH** | **IL6** | **AKT1** | **VEGFA** | **TNF** | **EGFR** | **SRC** | **MAPK3** | **CASP3** | **JUN** |
| --- | --- | --- | --- | --- | --- | --- | --- | --- | --- | --- |
| **cut-off value** | 163.306 | 104.347 | 170.791 | 92.7904 | 150.85 | 158.165 | 135.424 | 163.713 | 103.538 | 107.627 |
| **saikosaponin F** | 166.772 | \ | \ | \ | \ | \ | 199.014 | \ | \ | \ |
| **clemastanin B** | 189.414 | \ | 189.326 | \ | \ | 173.073 | 182.902 | 196.043 | 135.792 | \ |
| **kaempferol-3-*O*-rutinoside** | \ | \ | 191.354 | \ | \ | 169.085 | 171.068 | 168.899 | 146.313 | \ |
| **quercetin-3-*O*-robinobioside** | \ | \ | 191.566 | \ | \ | 161.709 | 171.02 | 164.312 | 148.423 | \ |
| **rutin** | \ | \ | 191.192 | \ | \ | 174.565 | 161.293 | 183.549 | 149.708 | \ |
| **indigoticoside A** | \ | \ | 176.66 | \ | \ | 159.685 | 158.383 | \ | 146.02 | \ |
| **saikosaponin A** | 171.374 | \ | \ | \ | \ | \ | 158.205 | \ | 129.496 | \ |
| **4,5-dicaffeoylquinic acid** | \ | \ | 179.639 | 120.228 | \ | 161.566 | 158.063 | 168.775 | 146.289 | \ |
| **narcissin** | \ | \ | 188.015 | \ | \ | 185.727 | 155.589 | \ | 146.654 | \ |
| **(+)-isolariciresinol 9'-O-glucoside** | \ | 113.492 | \ | \ | 166.602 | 164.224 | 155.329 | \ | 151.739 | \ |
| **6''-O-acetylsaikosaponin A** | 172.526 | \ | \ | \ | \ | \ | 149.934 | \ | 134.046 | \ |
| **guamarolin** | \ | 117.761 | \ | 111.678 | \ | \ | 144.311 | \ | 118.692 | 139.221 |
| **hyperoside** | \ | 106.583 | \ | \ | \ | \ | 143.299 | \ | 146.807 | 124.277 |
| **kaempferol-3-*O*-rhamnoside** | \ | \ | \ | \ | \ | \ | 140.231 | \ | 140.711 | 130.587 |
| **isoquercitrin** | \ | 107.55 | \ | \ | \ | \ | 138.523 | \ | 139.533 | 129.018 |
| **pregomisin** | \ | \ | \ | 110.3 | \ | \ | 138.219 | \ | 111.947 | 112.519 |
| **schisandronic acid** | \ | \ | \ | \ | \ | \ | 138.041 | \ | 115.337 | \ |
| **scoparone** | \ | \ | \ | 117.326 | \ | \ | 136.997 | \ | 129.98 | \ |
| **kadsuric acid** | \ | \ | \ | 97.5622 | \ | \ | 136.44 | \ | 120.048 | \ |
| **vitexin** | \ | \ | \ | 94.9705 | \ | \ | 136.279 | \ | 128.588 | 138.975 |
| **saikosaponin B2** | 173.639 | \ | \ | \ | \ | \ | \ | \ | 105.224 | \ |
| **glycochenodeoxycholic acid** | \ | \ | \ | 116.249 | \ | \ | \ | \ | 121.139 | \ |

|  | **GAPDH** | **IL6** | **AKT1** | **VEGFA** | **TNF** | **EGFR** | **SRC** | **MAPK3** | **CASP3** | **JUN** |
| --- | --- | --- | --- | --- | --- | --- | --- | --- | --- | --- |
| **cut-off value** | 163.306 | 104.347 | 170.791 | 92.7904 | 150.85 | 158.165 | 135.424 | 163.713 | 103.538 | 107.627 |
| **hyocholic acid** | \ | \ | \ | 120.251 | \ | \ | \ | \ | 107.597 | \ |
| **chenodeoxycholic acid** | \ | \ | \ | 116.626 | \ | \ | \ | \ | \ | \ |
| **schisanlactone D** | \ | \ | \ | 115.312 | \ | \ | \ | \ | 109.437 | \ |
| **hyodeoxycholic acid** | \ | \ | \ | 110.508 | \ | \ | \ | \ | \ | \ |
| **chlorogenic acid** | \ | \ | \ | 102.266 | \ | \ | \ | \ | 108.262 | 115.462 |
| **capillarisin** | \ | \ | \ | 97.4097 | \ | \ | \ | \ | 108.019 | 125.909 |
| **quercetin** | \ | \ | \ | 94.0924 | \ | \ | \ | \ | \ | 119.479 |
